# Methanol-dependent *Escherichia coli* strains with a complete ribulose monophosphate cycle

**DOI:** 10.1038/s41467-020-19235-5

**Published:** 2020-10-26

**Authors:** Philipp Keller, Elad Noor, Fabian Meyer, Michael A. Reiter, Stanislav Anastassov, Patrick Kiefer, Julia A. Vorholt

**Affiliations:** 1 Institute of Microbiology, Department of Biology, ETH Zurich, 8093 Zurich, Switzerland; 2 Institute of Molecular Systems Biology, Department of Biology, ETH Zurich, 8093 Zurich, Switzerland

**Keywords:** Metabolic engineering, Biochemical reaction networks, Applied microbiology

## Abstract

Methanol is a biotechnologically promising substitute for food and feed substrates since it can be produced renewably from electricity, water and CO_2_. Although progress has been made towards establishing *Escherichia coli* as a platform organism for methanol conversion via the energy efficient ribulose monophosphate (RuMP) cycle, engineering strains that rely solely on methanol as a carbon source remains challenging. Here, we apply flux balance analysis to comprehensively identify methanol-dependent strains with high potential for adaptive laboratory evolution. We further investigate two out of 1200 candidate strains, one with a deletion of fructose-1,6-bisphosphatase (*fbp*) and another with triosephosphate isomerase (*tpiA*) deleted. In contrast to previous reported methanol-dependent strains, both feature a complete RuMP cycle and incorporate methanol to a high degree, with up to 31 and 99% fractional incorporation into RuMP cycle metabolites. These strains represent ideal starting points for evolution towards a fully methylotrophic lifestyle.

## Abstract (web summary)

The engineering of methanol-dependent growth in *Escherichia coli* is challenging. Here, the authors predict and experimentally validate methanol-dependent strains with a complete RuMP cycle and high potential for the development of a methylotrophic platform organism.

## Introduction

Current biotechnological processes are mainly fueled by plant-derived sugars. However, non-food and non-feed alternatives are gaining interest due to their independence from agricultural produce^1–4^. Methanol is a particularly promising alternative, as it can be produced sustainably from CO_2_^5–7^ in conjunction with renewable electricity or from methane^8–12^. Furthermore, methanol has the potential to replace fossil fuels as an energy storage medium and can be used as a platform chemical for a wide range of synthetic products. Because of its benefits, a large-scale transition of industrial processes to a methanol-based economy has been proposed^13–15^.

Biologically, methanol serves as a carbon and energy source for various microbes, the methylotrophs. Although natural methylotrophic organisms are well studied, their biotechnological applications remain limited^1,16^. An alternative to the industrial utilization of natural methylotrophs is the introduction of methylotrophy to biotechnologically well-established organisms with an already existing product portfolio, such as *Escherichia coli*^17^. This would enable the conversion of biotechnologically relevant strains to methylotrophs and would pave the way for sustainable non-food and non-feed-dependent bulk and specialized products. Apart from its potential for biotechnological applications, the generation of a synthetic methylotroph is of value because it would help to uncover and test basic design principles of methylotrophy.

This engineering challenge of creating a synthetic methylotrophic organism has attracted considerable interest in recent years, with the focus mostly on the naturally occurring ribulose monophosphate (RuMP) cycle due to its energy efficiency^18–31^. The construction of the RuMP cycle in *E*. *coli* theoretically requires the expression of only three genes, i.e., a methanol dehydrogenase (*mdh*), a 3-hexulose 6-phosphate synthase (*hps*) and a 6-phospho 3-hexuloisomerase (*phi*). However, while heterologous expression of *mdh*, *hps* and *phi* results in methanol incorporation into central carbon metabolites, growth on methanol alone is not possible due to a variety of reasons including enzyme expression levels, gene regulation and autocatalytic cycle constraints.

A possible way forward is the generation of methanol-dependent strains that require methanol assimilation together with a co-substrate for growth. This strategy leads to strains where natural selection favors improved methanol assimilation and thus provides ideal starting points for subsequent long-term adaptive laboratory evolution experiments. Gleizer et al.^32,33^ recently applied a similar approach to create a fully autotrophic *E*. *coli*, highlighting the power of the approach. Methanol-dependent strains have been described in literature^21,22,28,29^, which-due to a compromised RuMP cycle-lacked the potential for evolution towards growth on methanol alone.

In this study, we aim to engineer methanol-dependent *E. coli* strains that retain the ability to become fully methylotrophic. For this purpose, we develop an algorithm based on flux balance analysis (FBA) to predict suitable genetic backgrounds and performance metrics, and then select the most promising candidates for evolution. Using this approach, we predict 1200 methanol-dependent *E*. *coli* strains with a complete RuMP cycle and select two promising ones for experimental investigation. These strains need only a single gene deletion to couple biomass production to methanol assimilation and are predicted to significantly incorporate methanol into RuMP cycle intermediates. Indeed, both strains require methanol for growth in the presence of pyruvate and incorporate methanol into RuMP cycle intermediates to an extent that exceed that of previously reported methanol-dependent strains. As the strains retain a complete RuMP cycle, they are ideal for evolution towards a fully methylotrophic *E*. *coli* and, ultimately, a platform organism for sustainable applications in biotechnology.

## Results

### In silico prediction of methanol-dependent strains with a complete ribulose monophosphate cycle

**Fig. 1:**
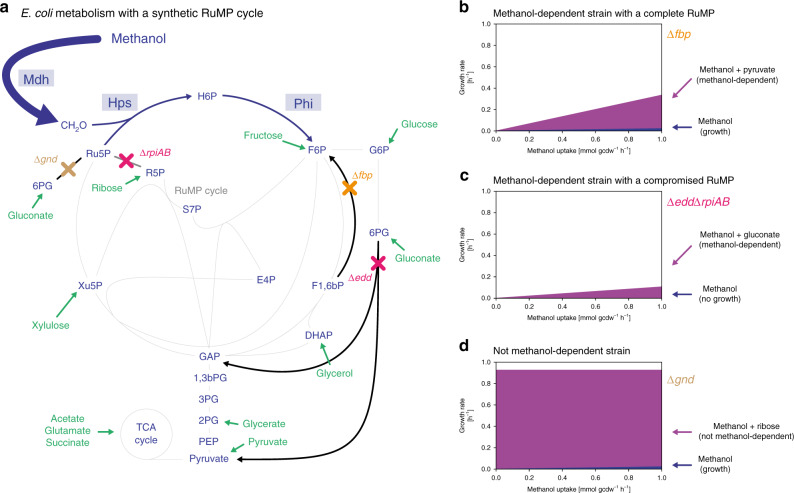
In silico prediction of methanol-dependent strains with the ribulose monophosphate cycle. **a** Central metabolism of *E. coli* including RuMP cycle gene products methanol dehydrogenase (Mdh), 3-hexulose 6-phosphate synthase (Hps) and 6-phospho 3-hexuloisomerase (Phi). Synthetic pathway reactions and enzymes are depicted in blue, endogenous metabolic reactions in gray and in silico-tested co-substrates in green. Genes deleted in **b**-**d** are indicated in orange; ∆*fbp* (**b**), pink; ∆*edd*∆*rpiAB* (**c**), and brown; ∆*gnd* (**d**). **b**–**d** Predicted growth rate in correlation with the methanol uptake rate of *E*. *coli* strains (∆*fbp*, ∆*edd*∆*rpiAB* and ∆*gnd*) on methanol and co-substrate (purple) and methanol alone (blue) in methanol-dependent strains (**b**), methanol-dependent strains with a compromised RuMP cycle (**c**) and not methanol-dependent strains with a complete RuMP cycle (**d**). Co-substrate uptake rate was set to 10 mmol gcdw^−1^ h^−1^. Abbreviations of genes and the encoding enzymes: *mdh*, methanol dehydrogenase; *hps*, 3-hexulose 6-phosphate synthase; *phi*, 6-phospho 3-hexuloisomerase; *rpiAB*, ribose phosphate isomerase A and B; *fbp*, fructose 1,6-bisphosphatase; *edd*, phosphogluconate dehydratase; *gnd*, 6-phosphogluconate dehydrogenase; MeOH, methanol; CH_2_O, formaldehyde; H6P, arabino 3-hexulose 6-phosphate; F6P, fructose 6-phosphate; G6P, glucose 6-phosphate; 6PG, 6-phosphogluconate; F1,6bP, fructose 1,6-bisphosphate; S7P, sedoheptulose 7-phosphate; E4P, erythrose 4-phosphate; R5P, ribose 5-phosphate; Ru5P, ribulose 5-phosphate; Xu5P, xylulose 5-phosphate; GAP, glyceraldehyde 3-phosphate; DHAP, dihydroxyacetonephosphate; 1,3bPG, 1,3-bisphosphoglycerate; 3PG, 3-phosphoglycerate; 2PG, 2-phosphoglycerate; PEP, phosphoenolpyruvate; RuMP cycle, ribulose monophosphate cycle; TCA cycle, tricarboxylic acid cycle.

Methanol-dependent strains require methanol for growth, as their biomass generation is stoichiometrically coupled to methanol assimilation. This coupling is achieved by introducing gene deletions that prevent the conversion of multi-carbon co-substrates into essential biomass precursors, which is rescued by concomitant utilization of methanol. To identify gene deletions that result in such strains, we modeled *E*. *coli* metabolism using FBA^34^. The underlying stoichiometric model was the *E. coli* core model^35^ to which the reactions of the Entner-Doudoroff pathway were added to better represent this basic metabolic flexibility of *E. coli*. Furthermore, we included the detoxification pathway for formaldehyde as it is a key methanol assimilation intermediate and the missing enzymes of the RuMP cycle, i.e. Mdh, Hps and Phi (Fig. 1a, see methods for detailed list). We queried this model and variants thereof—representing different genetic backgrounds of *E. coli* (see below)—for growth on methanol alone, on methanol together with a multi-carbon co-substrate or on co-substrate alone. The results were then filtered for candidates that grow in the first two conditions and not in the latter in order to obtain methanol-dependent strains that retain the potential for a methylotrophic lifestyle (Fig. 1b), and can thus evolve in a methanol co-substrate growth regime. This excluded metabolic makeups^21,22,28,29^ that lacked the potential for pure methylotrophic growth due to a compromised RuMP cycle (Fig. 1c).

As co-substrates, we limited our search to acetate, fructose, gluconate, glucose, glutamate, glycerate, glycerol, ribose, xylulose, pyruvate and succinate. These substrates broadly cover the major central metabolism entry points, including those of glycolysis, the pentose phosphate pathway, and the tricarboxylic acid (TCA) cycle (Fig. 1a).

To simulate different genetic *E. coli* backgrounds, model variants were generated by systematically deleting sets of reactions. We tested 33 single gene deletions and their combinations of up to size 5 (for details see Supplementary Data 1). In total, we analyzed 237,336 different genetic backgrounds and more than 2 million conditions. With this approach, 1200 methanol-dependent strains were identified, of which 12 had only a single knockout, and 57 required two knockouts (Supplementary Fig. 1a, Supplementary Data 3). Notably, most solutions were based on co-substrates that enter metabolism via gluconeogenesis (acetate, glutamate, glycerate, pyruvate, and succinate) (Supplementary Fig. 1b). Among these gluconeogenic co-substrates, acetate and glutamate were predicted to support the highest number of methanol-dependent strains. Strikingly, gluconate was the only C5/C6 co-substrate that yielded methanol-dependent solutions.

### Evaluation of methanol-dependent strains in silico

The number of 1200 predicted methanol-dependent strains (Supplementary Fig. 1) exceeds experimental validation capabilities. To select the most promising genetic backgrounds for subsequent adaptive evolution experiments, we developed two metrics by which we ranked all candidates.

**Fig. 2:**
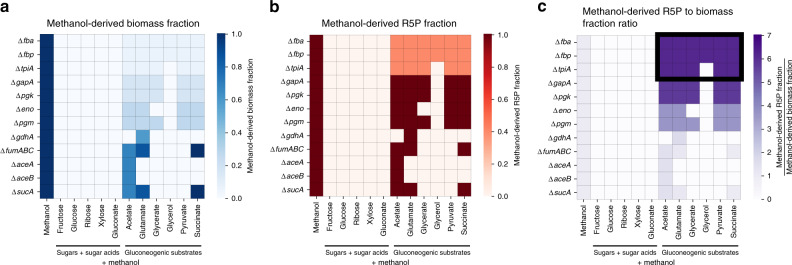
In silico validation of methanol-dependent strains. Methanol-dependent strains with gene deletions representing a single reaction knockout in FBA are shown. **a** The methanol-derived biomass fractions for predicted methanol-dependent strains growing on methanol with or without a sugar/sugar acid co-substrate, or a gluconeogenic co-substrate. The methanol-derived biomass fraction ranges from 0.0 (the biomass is methanol-independent) to 1.0 (the biomass fully depends on methanol). **b** The methanol-derived R5P fractions for predicted methanol-dependent strains. It ranges from 0.0 (completely independent) to 1.0 (completely dependent). **c** The ratio of the methanol-derived R5P fraction to the one of the biomass is used to compare the predicted methanol-dependent strains. The higher the ratio, the more promising the experimental implementation of the strain with an operational RuMP cycle (the top strains and conditions are highlighted by a black box). Source data are provided as a Source Data file.

First, we calculated the predicted methanol-derived biomass fraction for each genetic background (Fig. 2a, Supplementary Fig. 2). Assuming that an unevolved strain is unable to sustain high levels of methanol incorporation, suitable candidate strains require only small amounts of methanol incorporation into biomass when grown together with their co-substrates. For example, strains containing a deletion of any of the gluconeogenic genes encoding fructose 1,6-bisphosphate aldolase (*fbaAB*), 1,6-fructose bisphosphatase (*fbp*) or triosephosphate isomerase (*tpiA*) are expected to form 6.5% of their biomass from methanol. We considered this favorable for experimental implementation as it results in a low initial metabolic burden on the synthetic pathway.

Second, we aimed to determine the dependency of the RuMP cycle on methanol assimilation (Fig. 2b, Supplementary Fig. 3). Here, we used R5P as a proxy for the whole cycle and calculated its methanol-derived fraction, i.e., we determined which parts of the molecule are derived from methanol and which from the co-substrate. We reasoned that ideal candidate strains have a medium degree of methanol incorporation into RuMP cycle intermediates. Too little incorporation might lead to limited selective pressure towards better methanol assimilation in adaptive evolution experiments, while too high levels are unlikely feasible in an unevolved strain due to metabolic regulation and autocatalytic cycle constraints. Examples of promising strains with single gene deletions were *fbaAB, fbp* or *tpiA* with a predicted 40% incorporation of methanol into the RuMP cycle when grown with any of the tested gluconeogenic carbon sources. Another set of genetic backgrounds that are fully dependent on methanol for RuMP cycle operation were also found.

Ultimately, we combined both metrics and ranked all candidate strains by their predicted ratio of RuMP cycle methanol-dependency (i.e. methanol-derived R5P fraction) to methanol-derived biomass fraction, thereby optimizing both dimensions. The top strains, Δ*fbaAB*, Δ*fbp*, and Δ*tpiA*, required a single gene deletion and were coupled to a gluconeogenic co-substrate (Fig. 2c). Multiple knockout strains were generally found to be less promising due to their high demand for methanol assimilation.

### Experimental investigation of methanol-dependent growth

**Fig. 3:**
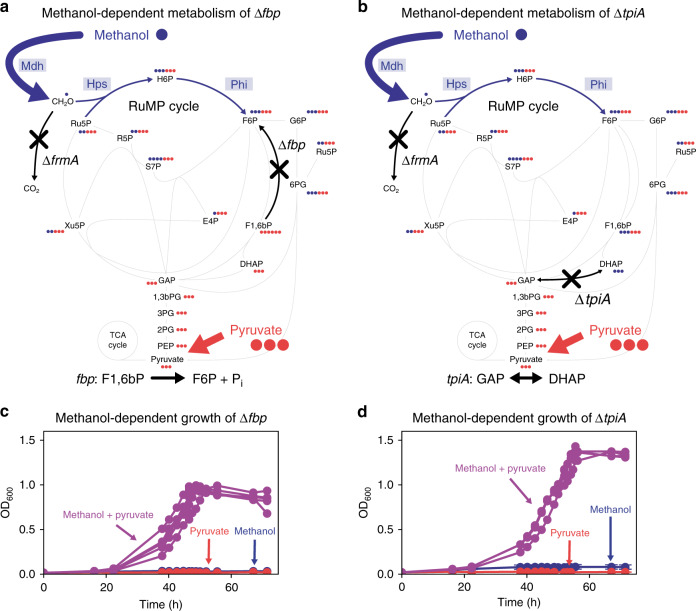
Experimental investigation of methanol-dependent growth. **a, b** Central metabolism of the methanol-dependent *E. coli* strains Δ*frmA*Δ*fbp* (**a**) and Δ*frmA*Δ*tpiA* (**b**) expressing the RuMP cycle genes *mdh*, *hps* and *phi*. Synthetic pathway reactions and enzymes are depicted in blue, the endogenous metabolic reactions are in gray and gene deletions are in black. The number of dots represents the number of carbons of the specific metabolite and the coloring. The dots are colored according to thepredicted methanol-derived metabolite fraction, whereby the part of the metabolite that stoichiometrically must originate from methanol is illustrated in blue, the one that is produced from pyruvate in red. **c, d** Methanol-dependent growth of Δ*frmA*Δ*fbp* (**c**) (*n* = 5) and Δ*frmA*Δ*tpiA* (**d**) (*n* = 4) expressing *Cupriavidus necator mdh*2 CT4-1 from pSEVA424 and *Methylobacillus flagellatus hps* and *phi* from pSEVA131. Methanol-dependent strains were cultivated in minimal medium supplemented with both methanol (500 mM) plus pyruvate (20 mM) (purple), methanol (500 mM) as the sole carbon source (blue) and pyruvate (20 mM) as the sole carbon source (red). The biological replicates in the multi-substrate condition (purple) are plotted separately. Error bars represent the standard deviation and are not visible because they are smaller than the size of the markers. For abbreviations, see Fig. 1. Source data are provided as a Source Data file.

Based on our in silico analyses, three genetic backgrounds (Δ*fbp* (Fig. 3a), Δ*tpiA* (Fig. 3b) and Δ*fbaAB*) were selected for further analysis. All three strains were predicted to be methanol-dependent under acetate, glutamate, glycerate, pyruvate, and succinate cultivation conditions and to rely on the disruption of gluconeogenesis to couple growth to methanol assimilation. To further investigate the experimental feasibility of the genetic backgrounds, the experimentally validated essential genome of *E. coli* was considered^36^. Since *fbaA* is essential, we focused on *fbp* and *tpiA* for the experimental validation of methanol-dependent growth. In addition to mutating *fbp* and *tpiA*, we introduced a deletion mutation in *frmA* to disrupt the formaldehyde detoxification pathway, as it was shown to positively affect synthetic methylotrophy by preventing the loss of carbon in the form of CO_2_^22^. The disruption of the formaldehyde detoxification pathway ensures that there is no flux through this pathway, which is in agreement with the FBA predictions. To enable methanol assimilation, the plasmids pSEVA424, encoding a Mdh from *Cupriavidus necator*^19^, and pSEVA131, encoding Hps and Phi from *Methylobacillus flagellatus*^22^, were introduced. We chose pyruvate for the experimental implementation because it is a central branching point in metabolism and is more oxidized than the average carbon in the cell biomass^37^, which is a feature that helps compensate for the highly reduced carbon source methanol. After initial recovery in lysogeny broth (LB) medium, both strains were transferred to minimal medium containing either methanol (500 mM), pyruvate (20 mM), or both (500 mM and 20 mM, respectively). The Δ*frmA*Δ*fbp* strain grew readily on methanol plus pyruvate, while it did not grow on pyruvate or on methanol alone, confirming its methanol-dependent growth (Fig. 3c). The Δ*frmA*Δ*tpiA* strain required yeast extract as a growth initiator, which could be omitted after two passages in a shake flask (Supplementary Fig. 4). Subsequently, the strain grew on pyruvate plus methanol but not on either of the substrates alone resulting in higher yields than the Δ*frmA*Δ*fbp* strain (Fig. 3d). In conclusion, both strains showed methanol-dependent growth, confirming the applicability of the in silico approach.

### Methanol incorporation into central metabolism during exponential growth

For both strains, in silico analysis predicted a methanol-derived R5P fraction of 40% and of 6.5% for biomass during the co-consumption of pyruvate and methanol, implying high methanol incorporation into RuMP cycle intermediates (Fig. 2b) and low methanol incorporation into the overall biomass (Fig. 2a). To specifically estimate methanol incorporation into the entire set of RuMP cycle intermediates (F6P, Ru5P, Xu5P, R5P, S7P, E4P, F1,6bP, DHAP, and GAP) and biomass precursors (e.g., 2PG, 3PG, and PEP), we determined the methanol-derived metabolite fraction for each intermediate and precursor. The fractions were calculated using the same method as that used for the methanol-derived R5P fraction (Eq. 2). Whereas significant formation of the RuMP cycle intermediates F6P, Ru5P, Xu5P, R5P, S7P, and E4P from methanol was predicted for both strains, formation of F1,6bP and DHAP from methanol was only predicted for strain Δ*frmA*Δ*tpiA*. The knockout of *tpiA* should result in the exclusive formation of DHAP from methanol and of GAP from pyruvate. Consequently, in the Δ*frmA*Δ*tpiA* strain, F1,6bP should originate equally from methanol and pyruvate since it is formed via the condensation of GAP and DHAP (see also Fig. 3a, b; high methanol incorporation is expected in metabolites with a high fraction of blue dots, and low incorporation is expected in metabolites with red dots).

**Fig. 4:**
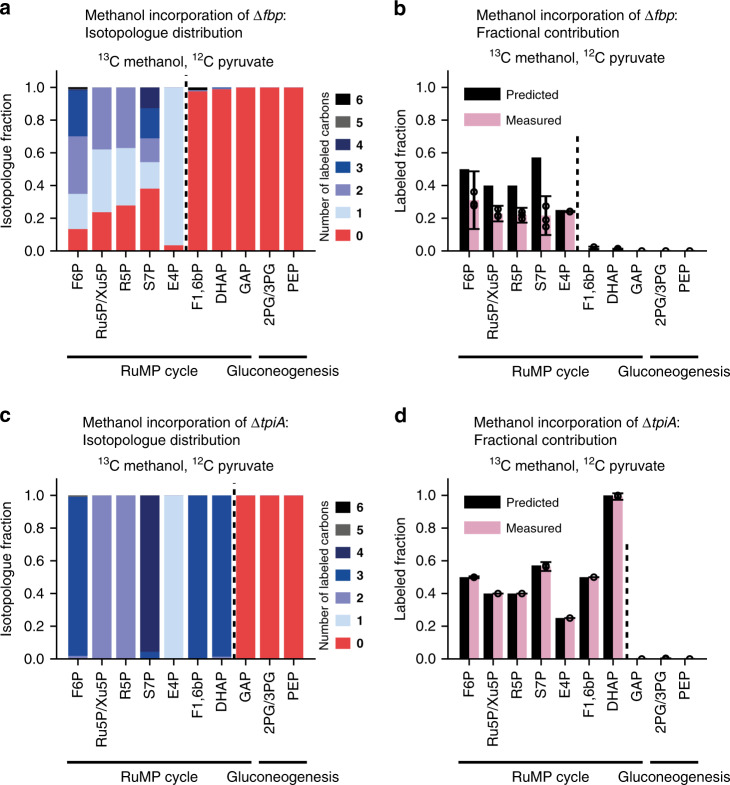
Methanol incorporation into central metabolism during exponential growth. **a**–**d** ^13^C isotope steady-state labeling experiment in Δ*frmA*Δ*fbp* (*n* = 3) and Δ*frmA*Δ*tpiA* (*n* = 5) expressing *Cupriavidus necator mdh*2 CT4-1 from pSEVA424 and *Methylobacillus flagellatus hps* and *phi* from pSEVA131. Methanol incorporation into the RuMP cycle and gluconeogenic metabolites is shown according to the isotopologue distribution (**a** for Δ*frmA*Δ*fbp*; **c** for Δ*frmA*Δ*tpiA*) and labeled fraction (**b** for Δ*frmA*Δ*fbp*; **d** for Δ*frmA*Δ*tpiA*). Cells were cultivated for at least 6 generations in minimal medium supplemented with ^13^C methanol (500 mM) and ^12^C pyruvate (20 mM) and sampled during exponential growth. A dashed line represents the location of the gene deletion (Δ*fbp* or Δ*tpiA*) in the metabolism; the metabolites on the left side of the line are expected to partially originate from methanol, while those on the right side are expected to be unlabeled (see also Fig. 3a, b for expected methanol incorporation into the measured metabolites). The predicted labeled fraction was based on the calculation of the methanol-derived metabolite fraction, which was calculated analogously to the methanol-derived R5P fraction (Eq. 2). The measured RuMP cycle metabolites were F6P, Ru5P/Xu5P, R5P, S7P, E4P, DHAP, GAP, and F1,6bP, and the measured gluconeogenic metabolites were 2PG/3PG and PEP. Ru5P/Xu5P and 2PG/3PG were not separable with the applied LC-MS method. The F6P isotopologue distribution and labeled fraction were calculated from UDP and UDP-glucose. E4P was detected in only 2 out of 5 replicates of strain Δ*frmA*Δ*tpiA*. Error bars represent the standard deviation. For abbreviations, see Fig. 1. Source data are provided as a Source Data file.

To verify the FBA predictions, an isotope steady-state labeling experiment was performed, in which cells grown on ^13^C methanol (500 mM) and ^12^C pyruvate (20 mM) were sampled in steady-state conditions during the exponential growth phase. As predicted (Fig. 3), the methanol-derived metabolites consistently incorporated labeled carbon from methanol, whereas the pyruvate-derived metabolites were formed from unlabeled pyruvate (Fig. 4). In the case of the strain Δ*frmA*Δ*tpiA*, the stoichiometric predictions exactly matched the values determined for the labeled fractions for all measured compounds (Fig. 4d, predicted vs. measured). All ribulose monophosphate intermediates contained labeled carbon, and some contained up to four labeled carbons (Fig. 4c, d). Strikingly, half of F6P was formed from methanol and DHAP even fully originated from methanol. Moreover, all metabolites further downstream of GAP in glycolysis were fully unlabeled. In the case of strain Δ*frmA*Δ*fbp*, the predicted methanol incorporation into RuMP cycle intermediates was significantly overestimated by FBA (Fig. 4b), where the average RuMP cycle metabolites labeled fraction was measured to be only (18 ± 3%), compared to 32% according to the model. This stands in contrast to strain Δ*frmA*Δ*tpiA*, which (exactly as predicted) incorporated approximately twice as much methanol (45% ± 0%), indicating that Δ*tpiA* could more efficiently reduce the flux from pyruvate into the RuMP cycle compared to Δ*fbp*. In addition, we confirmed the suitability of the strains for long-term evolution experiments in chemostat experiments. Here, methanol-dependent growth was sustained and the growth rates of both strains increased over time (Supplementary Fig. 6).

Overall, the results obtained emphasize the potential of both identified strains, Δ*frmA*Δ*fbp* and Δ*frmA*Δ*tpiA*, for long-term evolution experiments or further targeted engineering approaches towards synthetic methylotrophy.

## Discussion

Engineering organisms exhibiting synthetic methylotrophy remains a major challenge. Here, we use the strength of FBA to predict not only growth on a non-native carbon source but also the degree to which carbon can be assimilated into various central metabolites. To select promising methanol-dependent strains, we predicted methanol incorporation into the RuMP cycle and biomass to provide an estimate of the metabolic burden on the synthetic pathway for a strain. In total, we determined predictions for 1200 methanol-dependent strains with up to five gene knockouts and a wide range of metabolic dependencies on methanol assimilation, which will serve as a valuable resource for further investigation. For experimental validation, we focused on two single knockout strains (Δ*fbp* and Δ*tpiA*), which were generated in a Δ*frmA* background with a predicted methanol-derived R5P fraction of 40% and a biomass fraction of 6.5%. Indeed, our experimental validation showed that in the case of the Δ*frmA*Δ*tpiA* strain, approximately half of all carbon in the RuMP cycle intermediates was acquired from methanol. The high degree of methanol incorporation suggests that the recombinant RuMP cycle is indeed operational. Furthermore, strains with similar or even higher methanol-derived R5P and biomass fractions might be worth testing experimentally. For example, strains with deletions of either *gapA* or *pgk* are expected to perform the entire RuMP cycle with methanol while only requiring the generation of 17.5% of the biomass from methanol. Accordingly, the burden on the synthetic pathway is expected to be 2.7 times higher than that of the Δ*frmA*Δ*tpiA* strain, and thus it is yet another promising candidate to explore further in future work.

Beyond determining the origin of the biomass precursors via fractional labeling, the ^13^C tracer allows an in-depth analysis of the operating fluxes. In fact, our experimental validation showed that despite having the same predicted methanol incorporation into RuMP cycle intermediates and biomass (Fig. 4b, d, predicted), the strains Δ*frmA*Δ*fbp* and Δ*frmA*Δ*tpiA* resulted in striking differences. Δ*frmA*Δ*fbp* showed a distributed isotopologue pattern with zero- to four-fold labeled RuMP cycle metabolites (Fig. 4a), whereas Δ*frmA*Δ*tpiA* RuMP intermediates had a distinct pattern with only one isotopologue and three unlabeled carbons per metabolite (Fig. 4c). The labeling in the Δ*frmA*Δ*tpiA* strain matched the predictions, suggesting that the predicted fluxes in the RuMP cycle can reproduce the in vivo situation. In contrast, the analysis of the Δ*frmA*Δ*fbp* strain suggests a different flux distribution in the RuMP cycle than the one predicted by the metabolic model. The applied model utilizes the conventional RuMP cycle variant which employs transaldolase; however, alternative RuMP cycle variants might have to be considered to explain the methanol incorporation observed in the Δ*frmA*Δ*fbp* strain. In a recent study, it was shown that the overexpression of fructose-1,6-bisphosphatase 2 (*glpX*) activates the sedoheptulose bisphosphate variant^26^. Although *glpX* is not overexpressed in the Δ*frmA*Δ*fbp* strain, residual expression might be sufficient to activate the variant. In this RuMP variant, DHAP and E4P are converted to S7P over sedoheptulose 1,7-bisphosphate (S1,7bP), suggesting that the elevated availability of DHAP and E4P is required to drive the pathway. In both strains, E4P is formed similarly via transketolase (~25% labeled fraction in both strains); however, DHAP is produced entirely from pyruvate in the Δ*frmA*Δ*fbp* strain (1 ± 0% labeled fraction) and from methanol in the Δ*frmA*Δ*tpiA* strain (99 ± 2% labeled fraction). As pyruvate uptake is more efficient than methanol assimilation, the availability of DHAP is expected to be higher in the Δ*frmA*Δ*fbp* strain, which could activate the sedoheptulose bisphosphate pathway and explain the deviation from the metabolic model in the Δ*frmA*Δ*fbp* strain. The different flux distribution might also induce the more efficient methanol and pyruvate co-consumption of strain Δ*frmA*Δ*tpiA*, which achieved a significantly higher yield than Δ*frmA*Δ*fbp* (Fig. 3c, d). This could be explained by an increased CO_2_ production in strain Δ*frmA*Δ*fbp* since no other significant product formation was observed after complete pyruvate consumption (Supplementary Fig. 5).

To generate a fully synthetic methylotrophic *E*. *coli* strain, additional limiting metabolic factors must be identified and resolved, including the synthetic pathway efficiency, metabolic regulation and autocatalytic cycle constraints. To improve synthetic pathway efficiency, methanol-dependent strains such as Δ*frmA*Δ*fbp* and Δ*frmA*Δ*tpiA* provide an ideal screening background to test different RuMP cycle gene variants, as their growth rate depends directly on methanol assimilation efficiency. Hence, monitoring growth can serve as a direct read-out for the efficiency of synthetic pathways in vivo. Metabolic mis-regulation and autocatalytic cycle constraints are complex problems^38,39^, and we currently lack a targeted engineering strategy that can overcome them. This has been affirmed by a recent study in which an autotrophic *E*. *coli* was achieved. Studying several parallel lineages revealed that at least 11 mutations were required to enable the autotrophic phenotype whereby most mutations likely target metabolic regulation and autocatalytic cycle constraints. Since the RuMP cycle and the Calvin cycle show substantial overlap, applying ALE to methanol-dependent ancestor strains with a complete RuMP cycle is a promising strategy, and hence, the two strains represent ideal starting points for long-term chemostat evolution experiments.

Synthetic methylotrophy over the RuMP cycle bears great potential for biotechnology as it compares favorably with the recently successfully implemented reductive glycine pathway^40^ with respect to energy demand^18,41^. Apart from a higher ATP yield per assimilated unit of methanol, the RuMP cyle does not require a CO_2_-enriched atmosphere and is thus highly promising for future synthetic methylotrophy applications. During the revision of this paper, a related study was published that reports growth on methanol by *E*. *coli* using the RuMP cycle^31^. Chen et al. employed a different approach from what described here, in which the authors first designed a methanol-dependent strain with an incomplete RuMP cycle, conducted an ALE experiment, then fixed the RuMP cycle by expressing the previously deleted genes and evolved it further after applying kinetic modeling and targeted engineering. The final strain grew in the presence of methanol; however, it remains unclear if it builds up its entire biomass from methanol. Labeling experiments are required to exclude potential confounding incorporation from the multicarbon compounds provided with the medium in addition to methanol. The Δ*frmA*Δ*fbp* and Δ*frmA*Δ*tpiA* strains described here depend on methanol for growth and maintain the potential to rely exclusively on methanol as the carbon and energy source. Especially the for a long-term evolution promising strain Δ*frmA*Δ*tpiA* shows significant methanol incorporation into RuMP cycle intermediates that exceeds previously reported strains under steady-state conditions^21,22,28,29^. A future long-term evolution with the Δ*frmA*Δ*tpiA* or any of the other strains predicted here is a highly promising strategy to achieve exclusive growth on methanol and represents an important step towards a biotechnologically applicable methanol to product conversion.

## Methods

### Reagents and media

^13^C methanol (99%) was purchased from Cambridge Isotope Laboratories. All other chemicals were obtained from Sigma-Aldrich unless otherwise stated. Minimal M9 medium for bacterial cultivation consisted of salts (g L^−1^): Na_2_HPO_4_ (6.780), KH_2_PO_4_ (3.000), NaCl (0.500), NH_4_Cl (1.000), MgSO_4_·7H_2_O (0.490), CaCl_2_·2H_2_O (0.015) and trace elements (mg L^−1^): FeSO_4_·7H_2_O (0.516), ZnSO_4_·7H_2_O (0.090), CuSO_4_·5H_2_O (0.089), CoCl_2_·6H_2_O (0.091), MnSO_4_·H_2_O (0.061). Antibiotics, if required for plasmid propagation or cloning, were added in the following concentrations (mg L^−1^): ampicillin (100), kanamycin sulfate (50), streptomycin sulfate (20). Oligonucleotides used in this study are listed in Supplementary Data 6.

### Strain construction

The strains used in this study are listed in Supplementary Data 6. All strains originated from *E. coli* strain BW25113, which is the parental strain of the Keio collection^42^. Knockout strains were generated by applying a P1 transduction based protocol^43^. Single deletion strains from the Keio collection with kanamycin resistance cassette in the deleted gene were used as donor strains to exchange the target gene by a kanamycin resistance cassette. To generate multi-gene deletions, the kanamycin resistance cassette in the first deleted gene was excised by a FLP recombinase based pCP20 plasmid system^44^, which resulted in a kanamycin-sensitive strain, enabling the deletion of the second gene. All strains were verified by PCR amplification of the genomic target region using primers (Supplementary Data 6) that bind before and after the target gene and by Sanger sequencing (Microsynth AG, Switzerland).

### Plasmid construction

The plasmids used in this study are listed in Supplementary Data 6. To obtain pSEVA424 *mdh*2 CT4-1 *Cupriavidus necator*, the coding sequence of methanol dehydrogenase 2 (*mdh*2) variant CT4-1 from *Cupriavidus necator*^19^ was synthesized by Eurofins and introduced into pSEVA424. The plasmid pSEVA131 3-hexulose 6-phosphate synthase (*hps*) and 6-phospho 3-hexuloisomerase (*phi*) was constructed in a previous study^22^. The plasmids were confirmed by PCR amplification and by Sanger sequencing (Microsynth AG, Switzerland).

### In silico prediction and validation of methanol-dependent strains

Cobra python^45^ was used for flux balance analysis (FBA). The python version was 3.7.1. Executed scripts are accessible from ETH gitlab (https://gitlab.ethz.ch/kellerp/methanoldependentecoli). To perform FBA on the core metabolism of *E*. *coli*, the *E*. *coli* core model^35^ from BiGG^46^ was used. The following reactions were manually added to the model (-> irreversible, <-> reversible):

1. Exchange and transport reactions for methanol, 6-phosphogluconate (gluconate), ribose 5-phosphate (ribose), xylulose 5-phosphate (xylulose), 2-phosphoglycerate (glycerate).
2. phosphogluconate dehydratase (EDD): 6_pgc_c -> h2o_c + 2ddg6p_c.
3. 2-dehydro-3-deoxyphosphogluconate aldolase (EDA): 2ddg6p_c -> g3p_c + pyr_c.
4. methanol dehyrogenase (MEDH): methanol_c + nad_c < -> formaldehyde_c + nadh_c.
5. 3-hexulose 6-phosphate synthase (H6PS): ru5p__D_c + formaldehyde_c < -> hexulose6p_c.
6. 6-phosphate 3-hexuloisomerase (H6PI): hexulose6p_c < -> f6p_c.
7. formaldehyde dehydrogenase (FRMA): formaldeyhde_c + nad_c -> for_c + nadh_c.
8. formate dehydrogenase (FDH): for_c + nad_c -> co2_c + nadh_c.

To generate knockout candidates, we used standard constraint-based methods for synthetic auxotrophy based on flux-balanace analysis^32,34,47,48^. The following single reaction knockouts were combined for up to five knockouts: FBP, TPI, FBA, RPI, EDD + EDA, RPE, TALA, TKT1, TKT2, SUCDi, AKGDH, ICL, ME1 + ME2, PYK, PGK, PGM, ENO, GAPD, PFK, PGI, PGL, GND, PPS, PPCK, FUM, PDH, MALS, PFL, MDH, SUCOAS, GLUDy, NADHTRHD, FRMA. For each knockout combination, FBA was performed with methanol as sole carbon source (control) or with additional co-substrate (fructose: fru_e; glucose: glc__D_e; glycerol: dhap_e; ribose: r5p_e; xylulose: xu5p__D_e; gluconate: 6pgc_e; acetate: ac_e; glutamate: glu__L_e; glycerate: 2pg_e; pyruvate: pyr_e; succinate: succ_e).

The methanol-derived biomass and R5P fractions were calculated according to the Eqs. (1) and (2). For both equations, two conditions are compared. Methanol is provided as the sole carbon source in the first condition while methanol and pyruvate are supplied as carbon sources in the second condition. In both conditions, the production rate of biomass (Supplementary Fig. 2) or R5P (Supplementary Fig. 3) was calculated. The chosen objective function of the FBA model defined the target, for which the methanol-derived fraction was predicted. The objective function of the model was biomass production for the calculation of the methanol-derived biomass fraction and r5p_c production for the calculation of the methanol-derived R5P fraction. The methanol-derived fractions resulted from the ratio of the production rate in condition 1 to the one in condition 2. The HPS flux was used as the bottleneck reaction and set to 1 mmol gcdw^−1^ h^−1^, the methanol and co-substrate influx were set to maximal 1000 mmol gcdw^−1^ h^−1^.

1 $${\mathrm{methanol}} - {\mathrm{derived}}\,{\mathrm{biomass}}\,{\mathrm{fraction}} = \frac{{{\mathrm{biomass}}\,{\mathrm{production}}_{{\mathrm{condition}}\,{\mathrm{1}}}}}{{{\mathrm{biomass}}\,{\mathrm{production}}_{{\mathrm{condition}}\,{\mathrm{2}}}}}$$

2 $${\mathrm{methanol}} - {\mathrm{derived}}\,{\mathrm{R5P}}\,{\mathrm{fraction}} = \frac{{{\mathrm{R5P}}\,{\mathrm{production}}_{{\mathrm{condition}}\,{\mathrm{1}}}}}{{{\mathrm{R5P}}\,{\mathrm{production}}_{{\mathrm{condition}}\,{\mathrm{2}}}}}$$

The ratio between the two measures was calculated by dividing the methanol-derived R5P fraction by the one of the biomass.

The following procedure was applied to generate Fig. 2:

1. The modified *E*. *coli* core model, the selected single gene reactions (33 reactions), the co-substrates (11 substrates) and the maximal number of knockouts (up to 5) were defined.
2. The Hps reaction was defined as the limiting reaction.
3. Generation of the total strain space by combination of the single reaction knockouts for up to 5 knockouts.
4. Selection of the methanol-dependent strains from the total strain space. The inverse value of the biomass production on methanol and co-substrate was calculated for each methanol-dependent strain and condition combination (Supplementary Data 2, the higher the value, the more dependent is biomass production on methanol).
5. Removal of the methanol-dependent strains with an incomplete RuMP cycle that do not possess the capability of sole growth on methanol and of inefficient strains, which can produce less biomass than the parental strain on methanol as sole carbon source. The remaining methanol-dependent strains contain a complete RuMP cycle and the ability to exclusively live from methanol. The methanol-derived biomass fraction was calculated for each strain and condition according to Eq. (1) (Fig. 2a, Supplementary Data 3). For the calculation, see Supplementary Fig. 2.
6. Calculation of the methanol-derived R5P fraction for each methanol-dependent strain with a complete RuMP cycle according to Eq. (2) (Fig. 2b, Supplementary Data 4). For the calculation, see Supplementary Fig. 3.
7. Calculation of the ratio between the methanol-derived R5P fraction and the methanol-derived biomass fraction (Fig. 2c, Supplementary Data 5). The resulting ratio was used to classify the methanol-dependent strains by their potential for experimental implementation.

### Experimental investigation of methanol-dependent growth

The growth phenotype characterization of methanol-dependent strains was performed in a volume of 20 mL in 100 mL baffled shake flasks at 37 °C and 160 r.p.m. in a Minitron Incubator (Infors HT). All the used media contained Amp, Sm, isopropyl-β-d-thiogalactopyranosid (IPTG) (0.1 mM). IPTG was used to induce heterologous gene expression. Cryostocks of the strains ∆*frmA*∆*fbp* (*n* = 5) and ∆*frmA*∆*tpiA* (*n* = 4) expressing heterologous RuMP cycle genes were inoculated in 5 mL lysogeny broth (LB) in 14 mL Polypropylene Round Bottom Tubes (Falcon) and incubated overnight at 37 °C at 160 r.p.m. For the first passage in minimal medium, overnight grown LB cultures were transferred to minimal medium containing pyruvate (20 mM) and methanol (500 mM) by centrifugation (5000 *g*, 5 min, room temperature) of 1 OD unit (1 mL of OD_600_ = 1.0), discard of the supernatant and resuspension in minimal medium. The cells were grown until mid-exponential growth phase and 1 OD unit was transferred to fresh minimal medium by pipetting for the final growth phenotype characterization. In the final passage, each replicate was split into three conditions: pyruvate (20 mM), methanol (500 mM), and pyruvate (20 mM) plus methanol (500 mM). Methanol and pyruvate conditions were used as negative controls and growth was monitored by measuring the OD_600_. Supernatant sample analysis was performed by high performance liquid chromatography using an Ultimate 3000 UHPLC device (Thermo) equipped with a Rezex ROA Organic Acid H + column (7.8 × 300 mm; Phenomenex) as analytical column and a UV-detector (VWD 3400 RS detector). The mobile phase was 2.5 mM H_2_SO_4_ at a flow rate of 0.6 mL min^−1^ and the conditions were isocratic. The sample injection volume was 10 µL and the absorption at 190 nm was monitored for metabolite detection.

### ^13^C isotopic tracer analysis

The methanol incorporation predictions (Fig. 4b, d) were calculated analogous to the methanol-derived R5P fraction according to Eq. (2), the objective function was changed from r5p_c to the metabolite of interest.

The ^13^C isotopic tracer analysis of methanol-dependent strains was performed in a volume of 20 mL in 100 mL baffled shake flasks at 37 °C and 160 r.p.m. in a Minitron Incubator (Infors HT). The used media contained Amp, Sm, isopropyl-β-d-thiogalactopyranosid (IPTG) (0.1 mM). IPTG was used to induce heterologous gene expression. Cryostocks of the strains ∆*frmA*∆*fbp* (*n* = 3) and ∆*frmA*∆*tpiA* (*n* = 5) expressing heterologous RuMP cycle genes were inoculated in 5 mL LB in 14 mL Polypropylene Round Bottom Tubes (Falcon) and incubated overnight at 37 °C at 160 r.p.m. For the first passage, overnight grown LB cultures were transferred to minimal medium containing ^12^C pyruvate (20 mM) and ^13^C methanol (500 mM) by centrifugation (5000 *g*, 5 min, room temperature) of 1 OD unit (1 mL of OD_600_ = 1.0), discard of the supernatant and resuspension in minimal medium. Cells were grown until mid-exponential growth phase and 1 OD unit was transferred to fresh minimal medium for final ^13^C isotopic tracer analysis. Cells were again grown until mid-exponential growth phase and 1 OD unit was sampled for metabolome analysis. For the sampling, cells were applied to a polyethylene filter (0.2 µm) (prewashed with 50 °C warm ultra-pure water (MilliQ)) under vacuum and washed with 10 mL 37 °C warm ultra-pure water (MilliQ). After washing, filters were put into 8 mL precooled (−20 °C) quenching solution (60: 20: 20, acetonitrile: methanol: 0.1 M formic acid) and incubated for 10 min on ice to extract intracellular metabolites. After incubation, quenching solution was snap-frozen in liquid-nitrogen and lyophilized (−40 °C). Subsequently, dried samples were dissolved in ultra-pure water (MilliQ) and further diluted 1:5 in the starting conditions (230 µM tributylamine (TBA), 3% methanol, pH = 9) of the liquid chromatography-mass spectrometry (LC-MS) method to a biomass concentration of 100 ng/µL (assuming that 1 OD unit has 250 µg cell dry weight).

^13^C methanol incorporation into RuMP cycle and gluconeogenic metabolites was determined by nanoscale ion-pair reversed-phase high-performance liquid chromatography^49^ with a nano-2D Ultra LC system (Eksigent Technologies) coupled to a LTQ Orbitrap XL mass spectrometer (Thermo Fischer Scientific). Chromatographic separation was achieved by a C18 column (Reprosil-Gold 120 C18 3 µM, 0.1 × 100 mm, Dr. Maisch GmbH) as stationary phase and solvent A/B as mobile phase. Solvent A was 230 μM TBA at pH 9.0 and Solvent B 1:1 methanol:2-propanol. Solvent B was used as eluent with a multi-step gradient of 0 min, 0%; 35 min, 12%; 36 min, 90%; 48 min, 90%; 49 min, 0%; 60 min, 0% at a flow rate of 400 nL min^−1^. The sample injection volume was 1 µL. For mass-spectrometry, Fourier transform mass spectrometry (FTMS) was performed in negative mode with source settings set to a spray voltage of −2.1 kV, a capillary temperature of 250 °C, a capillary voltage of −28 V and a tube lens voltage of −80 V. Mass spectra were recorded as centroids at a resolution of 60'000 in full scan mode and a mass range of 100–1000 m/z with a scan rate of 1 Hz.

LC-MS measurements were analyzed by emZed2^50^. Metabolites were identified by m/z (mass tolerance of 0.003 mass units) and by retention time. Retention time was confirmed by commercially available standards. The peak area cut off was set at 10000/100 ng biomass. Isotopologue fractions (*s*_*i*_) and labeled fraction (LF) were calculated as described before^51^ by targeted peak integration of all detected isotopologues according to Eq. (3) and Eq. (4). Calculated values were corrected for natural ^13^C labeling.

3 $$s_i = \frac{{m_i}}{{\mathop {\sum }\nolimits_{{\mathrm{j}} = {\mathrm{0}}}^n m_j}}$$

4 $${\mathrm{LF}} = \frac{{\mathop {\sum }\nolimits_{{\mathrm{i}} = {\mathrm{0}}}^n m_i \ast {\mathrm{i}}}}{{{\mathrm{n}} \ast \mathop {\sum }\nolimits_{{\mathrm{i}} = {\mathrm{0}}}^n m_i}}$$

Here *m* is the abundance of an isotopologue, *n* the number of carbons in the metabolite and *i*, *j* the isotopologues.

Due to a contamination of the hexose phosphates peak in strain Δ*frmA*Δ*fbp*, fructose 6-phosphate isotopologue fractions (*s*_*F6P,i*_) and labeled fraction (LF_F6P_) were calculated from UDP and UDP-glucose labeling patterns by solving the corresponding linear equation system with least square.

### Chemostat evolution

The chemostat evolution experiments were performed in a 500 mL bioreactor (Infors-HT) at 37 °C, at 700 r.p.m. and aerated with compressed air. The pH was kept constant at 7.0 by the addition of 1 M NH_3_ or 1 M H_2_SO_4_ for strain Δ*frmA*Δ*fbp* and of 1 M NaOH and 1 M HCl for Δ*frmA*Δ*tpiA*. Culture volume (~400 mL) and dilution rate were determined by weighing the feed and waste medium vessels. Minimal medium for bacterial cultivation in the chemostast contained different trace elements to the ones used for growth phenotype and ^13^C isotopic tracer analysis (mg L^−1^): FeCl_3_·6H_2_O (0.500), ZnSO_4_·7H_2_O (0.090), CuSO_4_·5H_2_O (0.088), CoCl_2_·6H_2_O (0.091), MnCl_2_·H_2_O (0.045) for the strain Δ*frmA*Δ*fbp* and Na_2_ETDA (5.000), FeSO_4_·7H_2_O (1.000), ZnSO_4_·7H_2_O (1.000), CuSO_4_·5H_2_O (0.100), Co(NO_3_)_2_·6H_2_O (1.000), MnSO_4_·H_2_O (5.000), Na_2_MoO_4_·2H_2_O (0.100), NiCl_2_·6H_2_O (0.200) for the strain Δ*frmA*Δ*tpiA*. The reactor feed contained minimal medium supplemented with 500 mM methanol, 20 mM pyruvate, IPTG and appropriate antibiotics. The strain Δ*frmA*Δ*fbp* (*n* = 1) was evolved for 38 generations with a generation time of 25 h in the first 7 generations, 20 h for the next 6 generations and 15 h for the remaining generations. The strain Δ*frmA*Δ*tpiA* (*n* = 1) was evolved for 42 generations with a generation time of 39.5 h. Limiting pyruvate concentration was confirmed by HPLC. The growth phenotype determination (∆*frmA*∆*fbp*, *n* = 5; ∆*frmA*∆*tpiA* *n* = 4) and the ^13^C isotopic tracer analysis (∆*frmA*∆*fbp*, n = 5; ∆*frmA*∆*tpiA* *n* = 5) of the evolved methanol-dependent strains was performed as described above with the following exception: To avoid any selection while growth in LB medium, cryostocks of the evolved strains ∆*frmA*∆*fbp* and ∆*frmA*∆*tpiA* were not inoculated in 5 mL LB but were streaked-out on a minimal medium agar plate containing 500 mM methanol, 20 mM pyruvate, IPTG and appropriate antibiotics, which was incubated at 37 °C for several days. As soon as the cells reached a certain density on the plate, a loop full of bacteria was inoculated into the first passage of the liquid culture containing pyruvate (20 mM) and methanol (500 mM), from which the experiments were continued analogously as described above for the ancestral strains.

### Reporting summary

Further information on research design is available in the Nature Research Reporting Summary linked to this article.

## Supplementary information

Supplementary Information

Peer Review File

Reporting Summary

Description of Additional Supplementary Files

Supplementary Data 1

Supplementary Data 2

Supplementary Data 3

Supplementary Data 4

Supplementary Data 5

Supplementary Data 6

## Data Availability

Data supporting the findings of this work are available within the paper and its Supplementary Information files. A reporting summary for this Article is available as a Supplementary Information file. The datasets and materials generated and analyzed during the current study are available from the corresponding author upon request. The *E*. *coli* core model is accessible from the BIGG FBA model database (http://bigg.ucsd.edu/). Source data are provided with this paper.

## Source data

Source data

## Code availability

Scripts are accessible from ETH gitlab [https://gitlab.ethz.ch/kellerp/methanoldependentecoli].
