# Supplementary material for: Methanol-dependent Escherichia coli strains with a complete ribulose monophosphate cycle
Source: Nat Commun. 2020 Oct 26;11:5403. doi: 10.1038/s41467-020-19235-5 (PMC7588473; doi:10.1038/s41467-020-19235-5)
Supplement: Supplementary file 1 — Supplementary Information [file 41467_2020_19235_MOESM1_ESM.pdf]

Methanol-dependent *Escherichia coli* strains with a complete ribulose monophosphate cycle

Keller *et al.*

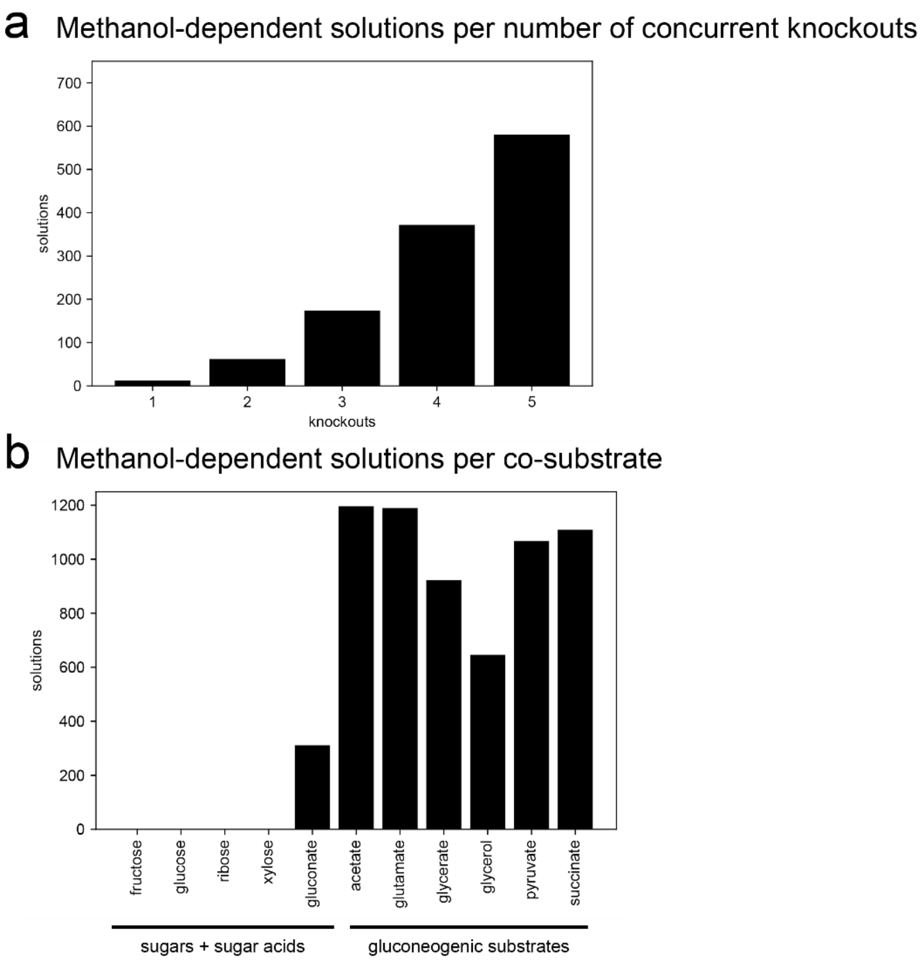

Supplementary Fig. 1. *In silico* prediction of methanol-dependent strains with ribulose monophosphate cycle. a-b Histograms of the 1200 methanol-dependent solutions (with up to 5 gene knockouts) that include the full RuMP cycle broken down by number of concurrent knockouts (a) or by the co-substrate (b). For abbreviations, see Fig. 1. Source data are provided as a Source Data file.

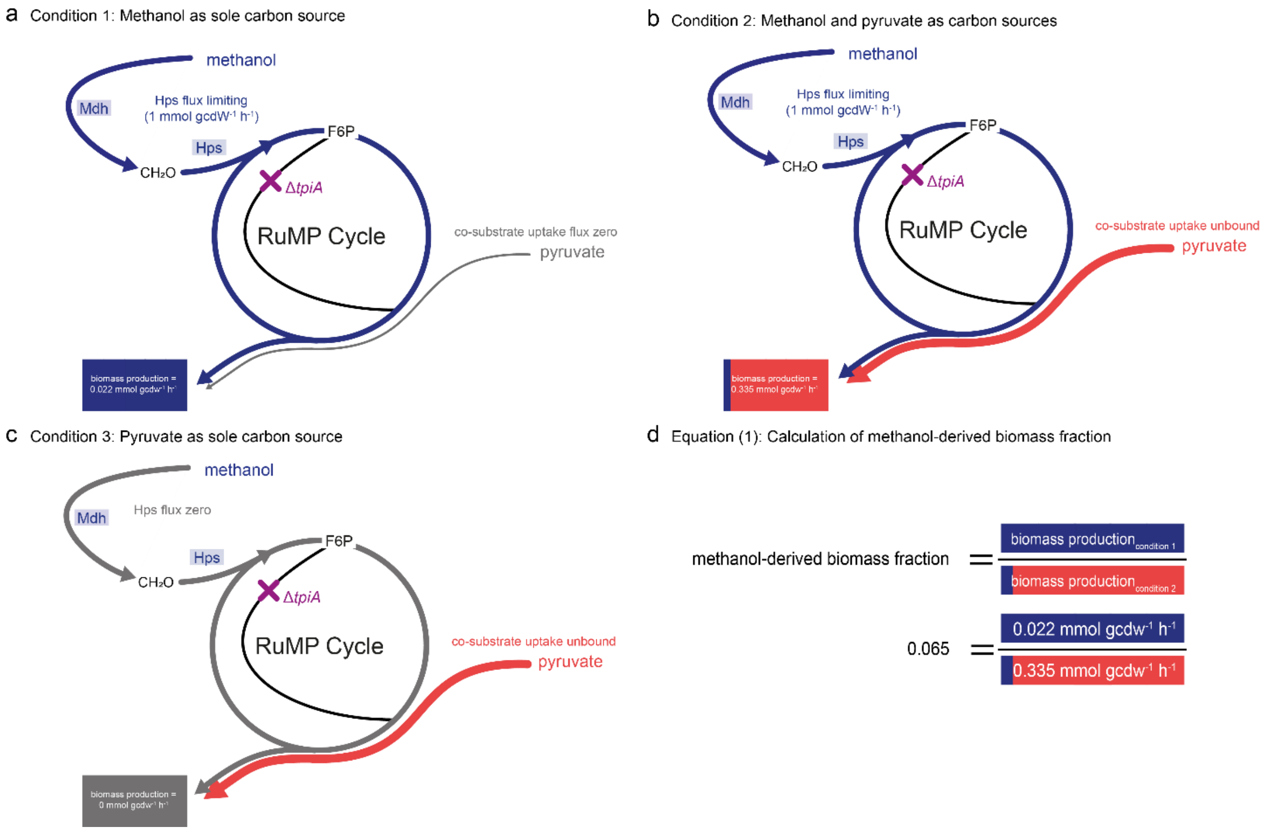

Supplementary Fig. 2. Calculation of the methanol-derived biomass fraction. a-c Central metabolism of the methanol-dependent strain Δ*tpiA* expressing the RuMP cycle genes *mdh*, *hps* and *phi*. The growth of the strain Δ*tpiA* is methanol-dependent on gluconeogenic substrates (here pyruvate). Three conditions were compared in respect to their biomass production: methanol as sole carbon source (a), methanol and pyruvate as carbon sources (b) and pyruvate as sole carbon source (c). The square in the background of biomass production shows the fraction of the biomass that originates from methanol in blue and from pyruvate in red. Condition c results in zero biomass formation because of its methanol-dependent production and the lack of provided methanol. Methanol-derived fluxes are depicted in blue, pyruvate-derived fluxes in red, inactive fluxes in gray, the RuMP cycle in black and the Δ*tpiA* gene deletion in purple. Methanol and pyruvate influx were, if the metabolite was present in the condition, provided in excess with a maximal flux of 1000 mmol $gcdw^{-1}$ h^{-1} and Hps flux was limited to 1 mmol $gcdw^{-1}$ h^{-1}. Arrow width qualitatively correlates to the by FBA predicted flux. **d** Calculation of the methanol-derived biomass fraction. The methanol-derived biomass fraction is calculated from the ratio between the biomass production in condition 1 over the one in condition 2.

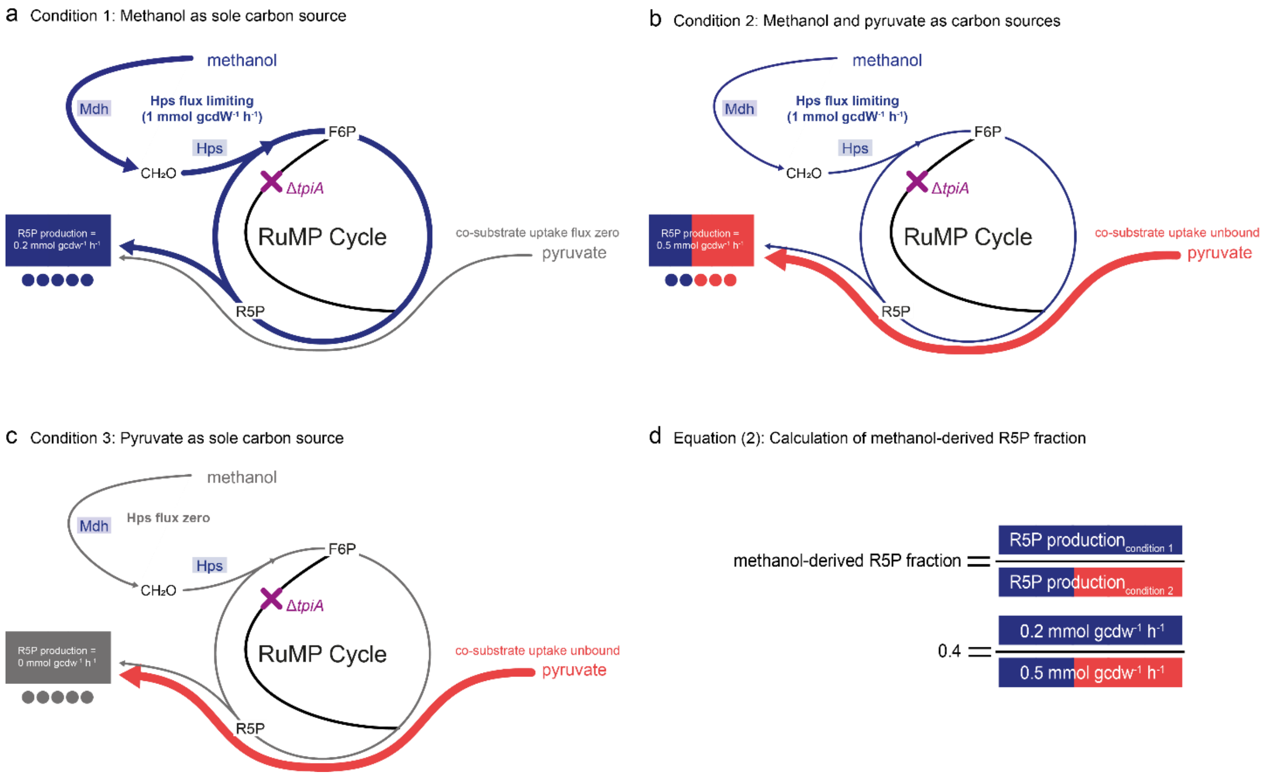

Supplementary Fig. 3. Calculation of the methanol-derived R5P fraction. a-c Central metabolism of the methanol-dependent strain Δ*tpiA* expressing the RuMP cycle genes *mdh*, *hps* and *phi*. The growth of the strain Δ*tpiA* is methanol-dependent on gluconeogenic substrates (here pyruvate). Three conditions were compared in respect to their R5P production: methanol as sole carbon source (a), methanol and pyruvate as carbon sources (b) and pyruvate as sole carbon source (c). The square in the background of R5P production and the five dots, representing the carbons of R5P, show the fraction of R5P that originates from methanol in blue and from pyruvate in red. Condition c results in zero R5P formation because of its methanol-dependent production and the lack of provided methanol. Methanol-derived fluxes are depicted in blue, pyruvate-derived fluxes in red, inactive fluxes in gray, the RuMP cycle in black and the Δ*tpiA* gene deletion in purple. Methanol and pyruvate influx were, if the metabolite was present in the condition, provided in excess with a maximal flux of 1000 mmol $gcdw^{-1}$ h^{-1} and Hps flux was limited to 1 mmol $gcdw^{-1}$ h^{-1}. Arrow width qualitatively correlates to the by FBA predicted flux. **d** Calculation of the methanol-derived R5P fraction. The methanol-derived R5P fraction is calculated from the ratio between the R5P production in condition 1 over the one in condition 2.

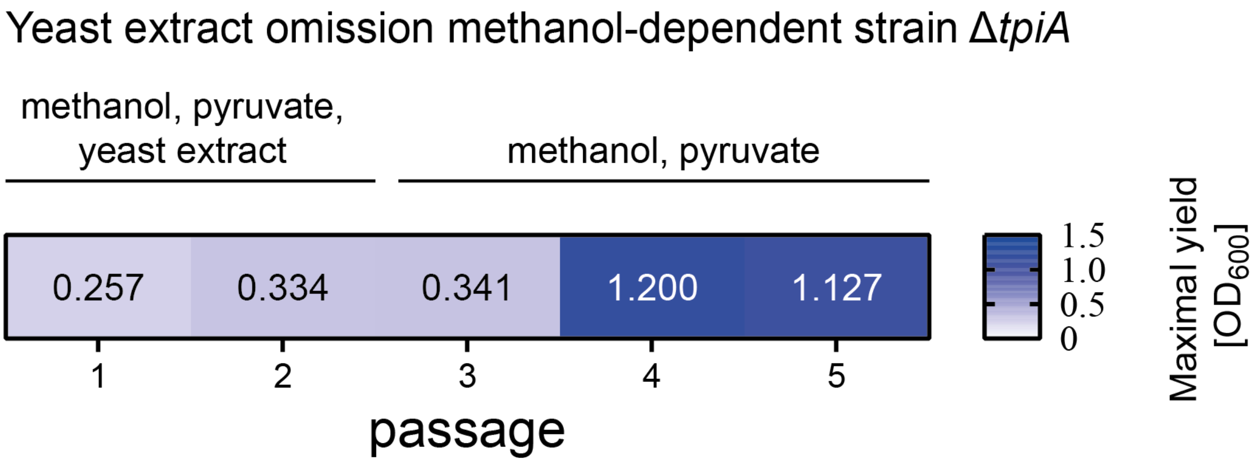

Supplementary Fig. 4. Adaptation of Δ*frmA*Δ*tpiA* to methanol-dependent growth without yeast extract. The methanol-dependent strain Δ*frmA*Δ*tpiA* expressing *Cupriavidus necator mdh*2 CT4-1 from pSEVA424 and *Methylobacillus flagellatus hps* and *phi* from pSEVA131 was adapted to grow without yeast extract. Methanol (500 mM), pyruvate (20 mM) and yeast extract (0.1 g/L) were present in the first two passages of shake-flask growth. Yeast extract was omitted in the third passage. Growth on methanol and pyruvate was optimized until the fifth passage to yield the final strain that was used in Fig. 3d, Fig. 4c and 4d. The color gradient (blue) represents the maximal yield in the respective passage. Source data are provided as a Source Data file.

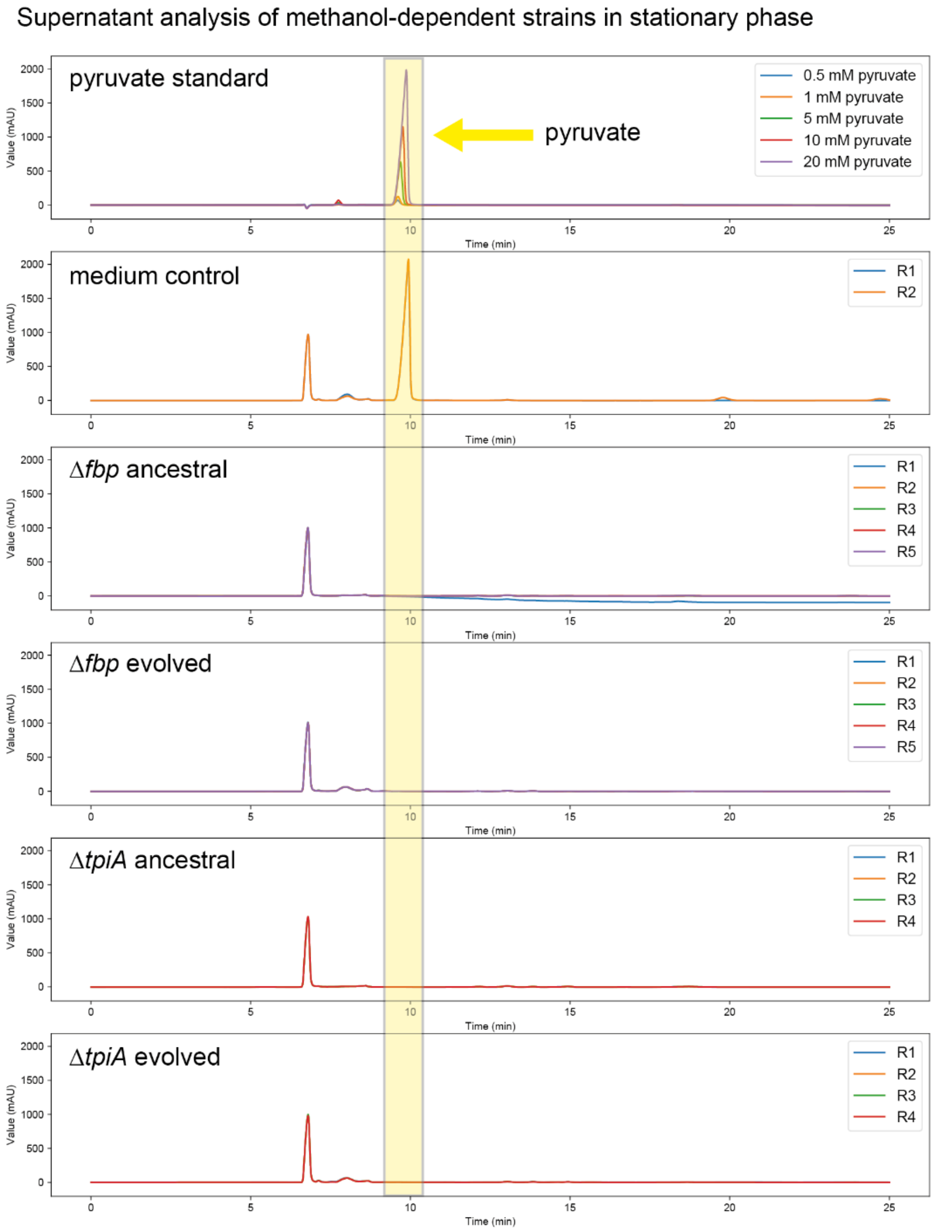

Supplementary Fig. 5. Supernatant analysis of the ancestral and evolved methanol-dependent strains Δ*fbp* and Δ*tpiA* in stationary phase. Supernatant samples originated from the growth experiment shown in Fig. 3 and Supplementary Fig. 6 and were taken during stationary phase. Methanol-dependent strains were cultivated in minimal medium supplemented with methanol (500 mM) and pyruvate (20 mM). Supernatant samples were analyzed by HPLC and metabolites were detected by monitoring the absorption at 190 nm. Source data are provided as a Source Data file.

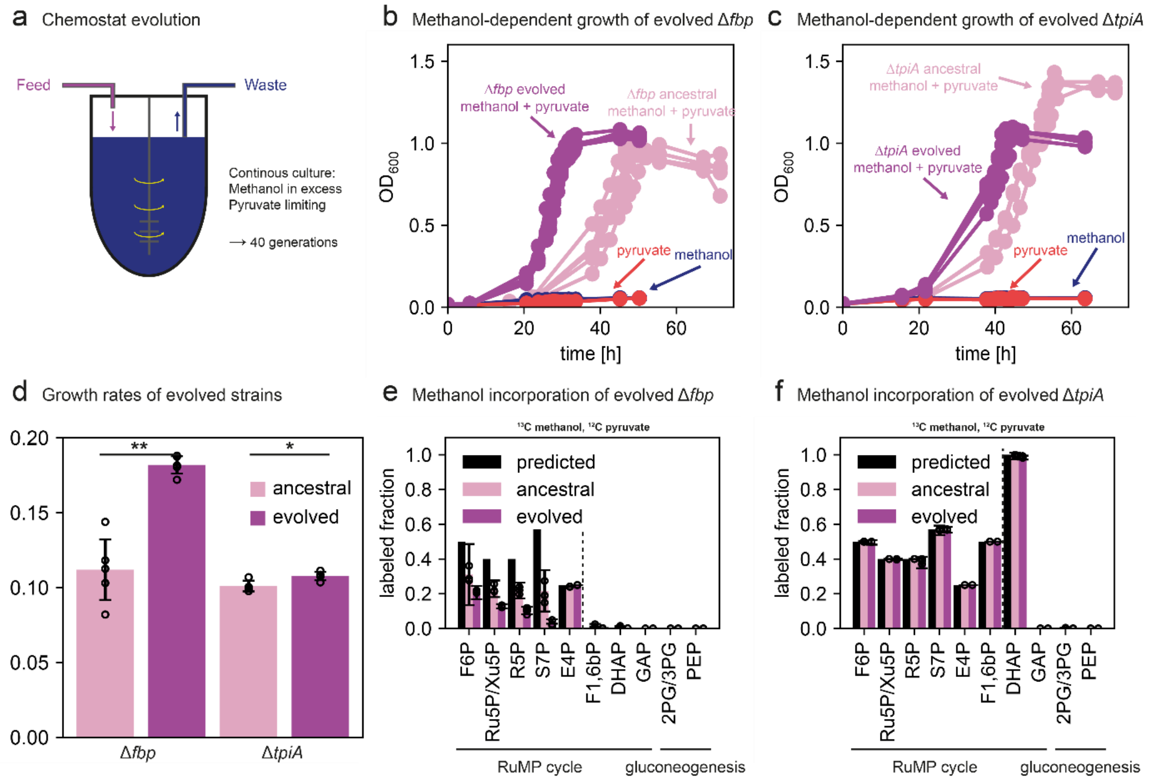

Supplementary Fig. 6. Chemostat evolution and characterization of the methanol-dependent strains Δ*frmA*Δ*fbp* and Δ*frmA*Δ*tpiA*. a The strains Δ*frmA*Δ*fbp* and Δ*frmA*Δ*tpiA* expressing *Cupriavidus necator mdh*2 CT4-1 from pSEVA424 and *Methylobacillus flagellatus hps* and *phi* from pSEVA131 were evolved in a chemostat setup. The reactor feed contained 500 mM methanol and 20 mM pyruvate and is indicated in purple. The reactor culture content and the waste efflux contained a limiting amount of pyruvate (below detection limit) and are depicted in blue. The strain Δ*frmA*Δ*fbp* (n = 1) was evolved for 38 generations with a generation time of 25 hours in the first 7 generations, 20 hours for the next 6 generations and 15 hours for the remaining generations. The strain Δ*frmA*Δ*tpiA* (n = 1) was evolved for 42 generations with a generation time of 39.5 hours. **b-c** Methanol-dependent growth of evolved Δ*frmA*Δ*fbp* (b) (n = 5) and evolved Δ*frmA*Δ*tpiA* (c) (n = 4). Methanol-dependent strains were cultivated in minimal medium supplemented with both methanol (500 mM) plus pyruvate (20 mM) (purple), methanol (500 mM) as the sole carbon source (blue) and pyruvate (20 mM) as the sole carbon source (red). The biological replicates in the multi-substrate condition (purple) are plotted separately. Error bars represent the standard deviation and are not visible because they are smaller than the size of the markers. **d** Growth rates of the ancestral (Fig. 3) and evolved methanol-dependent strains Δ*frmA*Δ*fbp* and Δ*frmA*Δ*tpiA*. Error bars represent the standard deviation and stars the significance of the difference, which was calculated by a two-sided Welch's unequal variances t-test (*p*-value = 0.0015 (Δ*frmA*Δ*fbp*); 0.0459 (Δ*frmA*Δ*tpiA*). **e-f** ^{13}C isotope steady-state labeling experiment of the ancestral (see also Fig. 4b and 4d) and evolved Δ*frmA*Δ*fbp* (n = 5) and Δ*frmA*Δ*tpiA* (n = 5). Methanol incorporation into the RuMP cycle and gluconeogenic metabolites is shown according to the labeled fraction (**e** for Δ*frmA*Δ*fbp*; **f** for Δ*frmA*Δ*tpiA*). Cells were cultivated for at least 6 generations in minimal medium supplemented with ^{13}C methanol (500 mM) and ^{12}C pyruvate (20 mM) and sampled during exponential growth. A dashed line represents the location of the gene deletion (Δ*fbp* or Δ*tpiA*) in the metabolism; the metabolites on the left side of the line are expected to partially originate from methanol, while those on the right side are expected to be unlabeled (see also Fig. 3a and 3b). The predicted labeled fraction was based on the calculation of the methanol-derived part of the metabolite, which was calculated analogously to the methanol-derived R5P fraction (Supplementary Fig. 2). The measured RuMP cycle metabolites were F6P, Ru5P/Xu5P, R5P, S7P, E4P, DHAP, GAP, and F1,6bP, and the measured gluconeogenic metabolites were 2PG/3PG and PEP. Ru5P/Xu5P and 2PG/3PG were not separable with the applied LC-MS method. The F6P isotopologue distribution and labeled fraction were calculated from UDP and UDP-glucose. E4P was detected in only 1 out of 5 replicates of the ancestral strain Δ*frmA*Δ*tpiA*. Error bars represent the standard deviation. For abbreviations, see Fig. 1. Source Data are provided as a Source Data file.
