# Supplementary material for: Methanol-dependent Escherichia coli strains with a complete ribulose monophosphate cycle
Source: Nat Commun. 2020 Oct 26;11:5403. doi: 10.1038/s41467-020-19235-5 (PMC7588473; doi:10.1038/s41467-020-19235-5)
Supplement: Supplementary file 3 — Reporting Summary [file 41467_2020_19235_MOESM3_ESM.pdf]

nature research

Corresponding author(s): Prof. Dr. Julia A. Vorholt

Last updated by author(s): *Sep 29, 2020*

Reporting Summary

Nature Research wishes to improve the reproducibility of the work that we publish. This form provides structure for consistency and transparency in reporting. For further information on Nature Research policies, see our [Editorial Policies](#) and the [Editorial Policy Checklist](#).

Statistics

For all statistical analyses, confirm that the following items are present in the figure legend, table legend, main text, or Methods section.

n/a	Confirmed	
☐	☒	The exact sample size (*n*) for each experimental group/condition, given as a discrete number and unit of measurement
☐	☒	A statement on whether measurements were taken from distinct samples or whether the same sample was measured repeatedly
☐	☒	The statistical test(s) used AND whether they are one- or two-sided<br>*Only common tests should be described solely by name; describe more complex techniques in the Methods section.*
☒	☐	A description of all covariates tested
☐	☒	A description of any assumptions or corrections, such as tests of normality and adjustment for multiple comparisons
☐	☒	A full description of the statistical parameters including central tendency (e.g. means) or other basic estimates (e.g. regression coefficient) AND variation (e.g. standard deviation) or associated estimates of uncertainty (e.g. confidence intervals)
☐	☒	For null hypothesis testing, the test statistic (e.g. *F*, *t*, *r*) with confidence intervals, effect sizes, degrees of freedom and *P* value noted<br>*Give P values as exact values whenever suitable.*
☒	☐	For Bayesian analysis, information on the choice of priors and Markov chain Monte Carlo settings
☒	☐	For hierarchical and complex designs, identification of the appropriate level for tests and full reporting of outcomes
☒	☐	Estimates of effect sizes (e.g. Cohen's *d*, Pearson's *r*), indicating how they were calculated

Our web collection on [statistics for biologists](#) contains articles on many of the points above.

Software and code

Policy information about [availability of computer code](#)

Data collection	Cobra python (Heirendt, L. et al. Creation and analysis of biochemical constraint-based models using the COBRA Toolbox v.3.0. Nat. Protoc. 14, 639–702 (2019)) was used to predict the dataset of the methanol-dependent strains with a complete RuMP cycle. The python version was 3.7.1. Executed scripts are accessible from ETH gitlab (https://gitlab.ethz.ch/kellerp/methanoldependentecoli).
Data analysis	Cobra python (Heirendt, L. et al. Creation and analysis of biochemical constraint-based models using the COBRA Toolbox v.3.0. Nat. Protoc. 14, 639–702 (2019)) was used to predict the fractional labeling of central metabolites of the methanol-dependent strains. EMZed2 (Kiefer, P. et al. eMZed: an open source framework in Python for rapid and interactive development of LC/MS data analysis workflows. Bioinformatics 29, 963–964 (2013)) was used for the analysis of the isotopic tracer experiment. Executed scripts are accessible from ETH gitlab (https://gitlab.ethz.ch/kellerp/methanoldependentecoli).

For manuscripts utilizing custom algorithms or software that are central to the research but not yet described in published literature, software must be made available to editors and reviewers. We strongly encourage code deposition in a community repository (e.g. GitHub). See the Nature Research [guidelines for submitting code & software](#) for further information.

Data

Policy information about [availability of data](#)

All manuscripts must include a [data availability statement](#). This statement should provide the following information, where applicable:

- Accession codes, unique identifiers, or web links for publicly available datasets
- A list of figures that have associated raw data
- A description of any restrictions on data availability

All data and materials are available upon request. The used E. coli core model is accessible from the BIGG FBA model database (http://bigg.ucsd.edu/).

Field-specific reporting

Please select the one below that is the best fit for your research. If you are not sure, read the appropriate sections before making your selection.

- [x] Life sciences
- [ ] Behavioural & social sciences
- [ ] Ecological, evolutionary & environmental sciences

For a reference copy of the document with all sections, see nature.com/documents/nr-reporting-summary-flat.pdf

Life sciences study design

All studies must disclose on these points even when the disclosure is negative.

Sample size	Sample sizes were chosen to reflect the state of the art in the field. Besides that no predetermination of the sample sizes was conducted. All experiments were performed in at least three biological replicates.
Data exclusions	No data were excluded from the analysis.
Replication	All experiments were performed in at least three biological replicates and all experiments were successfully replicated.
Randomization	No randomization was conducted.
Blinding	No blinding was conducted.

Reporting for specific materials, systems and methods

We require information from authors about some types of materials, experimental systems and methods used in many studies. Here, indicate whether each material, system or method listed is relevant to your study. If you are not sure if a list item applies to your research, read the appropriate section before selecting a response.

Materials & experimental systems

n/a	Involved in the study	
x		Antibodies
x		Eukaryotic cell lines
x		Palaeontology and archaeology
x		Animals and other organisms
x		Human research participants
x		Clinical data
x		Dual use research of concern

Methods

n/a	Involved in the study	
x		ChIP-seq
x		Flow cytometry
x		MRI-based neuroimaging
