# Supplementary material for: Methanol-dependent Escherichia coli strains with a complete ribulose monophosphate cycle
Source: Nat Commun. 2020 Oct 26;11:5403. doi: 10.1038/s41467-020-19235-5 (PMC7588473; doi:10.1038/s41467-020-19235-5)
Supplement: Supplementary file 4 — Description of Additional Supplementary Files [file 41467_2020_19235_MOESM4_ESM.pdf]

Description of Additional Supplementary Files

Supplementary Data 1
List of the tested 33 single gene deletions containing the corresponding reaction names in the FBA model, the reaction and the associated part of the metabolism.

Supplementary Data 2
Total solution space of predicted methanol-dependent strains predicted by the multi combinatorial FBA approach.

Supplementary Data 3
Methanol-dependent strains with a complete RuMP cycle and their methanol-derived biomass fraction under different co-consumption regime conditions predicted by the multi combinatorial FBA approach.

Supplementary Data 4
Methanol-dependent strains with a complete RuMP cycle and their methanol-derived R5P fraction under different co-consumption regime conditions predicted by the multi combinatorial FBA approach.

Supplementary Data 5
Methanol-dependent strains with a complete RuMP cycle and the ratio of R5P to biomass fraction under different co-consumption regime conditions predicted by the multi combinatorial FBA approach.

Supplementary Data 6
List of oligonucleotides, strains and plasmids used in this study.
