# Supplementary material for: Methanol-dependent Escherichia coli strains with a complete ribulose monophosphate cycle
Source: Nat Commun. 2020 Oct 26;11:5403. doi: 10.1038/s41467-020-19235-5 (PMC7588473; doi:10.1038/s41467-020-19235-5)
Supplement: Supplementary file 2 — Peer Review File [file 41467_2020_19235_MOESM2_ESM.pdf]

Reviewer #1 (Remarks to the Author):

The study of Keller et al. Methanol-dependent Escherichia coli strains with a complete ribulose monophosphate cycle" is interesting to read. The methodology is convincing and the iterative process for identifying the E. coli mutants is elegant.
But in my eyes there is a conceptional issue, the authors talk about methanol as a source for biomass production. However, if I understand the metabolic mode of the mutants, there is no incorporation of methanol in the biomass.
The authors reconstruct a methanol consuming pathway in E. coli. But the biomass production relies on pyruvate. On the other hand these substrates are not serving as a nitrogen source.
The authors do also not comment about the reason for the methanol autotrophy of these mutants. The Construction of a methanol dependent ribulose phosphate cycle is interesting, but the need to decouple the glycolysis at the level of aldolase or triose phosphate isomerase counteracts biomass production clearly.

Reviewer #2 (Remarks to the Author):

This is an excellent research paper that I really enjoyed reading it. I have to say that the scientific soundness, the significance of the content and the interest for the reader are high. However I am wondering if this paper fully satisfied both the criteria of novelty and advance in the field in regard to the following major point described below and previous studies in the field. This includes: one study ((doi.org/10.1038/s41589-020-0473-5) demonstrating the growth on methanol of a synthetic methylotroph and two studies (doi.org/10.1016/j.ymben.2017.11.016 & doi.org/10.1021/acssynbio.8b00093) addressing the importance of the full operation of the Rump Cycle on the methanol assimilation in synthetic methylotrophic strains. I advise the authors to discuss better those papers in regard to their results.

Major:

Point 1: The computational approach presented in this paper is interesting but I encourage the authors to perform more biological and computational validations to strengthen the novelty, the relevance and the genericity of the presented approach. For instance, does this approach reproduce the biological data obtain with previous methanol dependant strains with an incomplete RuMP cycle? Is this approach enough generic to be used to implement other non-native carbon source in E. coli? Here successful biological validations has been shown for 2 single K/O but what about the predicted double K/O? All these questions should be answered and discussed in the manuscript.

Point 2: The key point of the method presented here, i.e. the calculation of the ratios MRR and MBR is not trivial to understand. For instance, following the explanations given in both the results and methods sections, I understand that methanol influx was fixed at 1 mmol/gDCW/h and that co substrate influx varied between 0 and 1000). Based on that, I found that MRR could vary between 1 (Co substrate influx = 0, methanol influx = 1 and R5P production = 1) and 0.001 (Co substrate influx = 1000, methanol influx = 1 and R5P production = 1001 (I assume that R5P can also be produced by the co substrate, is it correct?)). Then in this case MRRn can vary between 0 and 1001 which is far to what you obtain (i.e. 0> MRRn> 1). I trust your method and I know that I definitely miss something but what? The raw data of the MRR and MBR for both the starting strain and the KO strains and eventually more explanations should be provided to help the reader.

Point 3: Authors argue that “ALE to methanol-dependent ancestor strains with a complete RuMP cycle is a promising strategy” to (I guess!) establish methylotrophy in E. coli but this should be demonstrated to really represent a significant advance in the field of synthetic methylotrophy.

Minor:
P2 L19-20: please add methanol in addition to the already written formate and CO2.
Fig 1B: I wonder if it is biologically possible to reach a such high growth rate (i.e. 1,5 h-1). What is the co-substrate uptake rate? Please add it on the figure. Is the growth dependent to the co substrate? Please add it in the text or in the figure.

P5 L110: What is the genotype of the wild type strain? If it's the strain containing the full RUMP cylce, then wild type is really not appropriate. This should be changed by starting strain.
Fig 2A & B: please correct: To my understanding both presented ratio are the normalised one so an underscript "n" should be added.
P7 L175: Please rephrase the sentence: " …methanol dependant for…" by methanol dependant under acetate….cultivation conditions.

Reviewer #3 (Remarks to the Author):

In this study, the authors develop methanol converting strains of E. coli based on the ribulose monophosphate cycle and utilizing flux balance analysis metabolic modeling. They experimentally built and profiled two strains, with fructose bisphosphatase and triosephosphate isomerase deletions, selected from ~1000 computationally-predicted strains that were high performing. They use a modified version of the core metabolic model from the BiGG database for the modeling component, which had the ED and RuMP cycle added. They used a recently published algorithm for predicted methanol-coupled growth designs. Experimental profiling included growth and 13C tracing measurements. The work is an interesting endeavor in computationally driven metabolic engineering, and the validation is promising. The use of a metabolic model of core metabolism only, rather than a genome-scale model, is the largest apparent practical concern, but as the authors included experimental validation, the importance of that concern is reduced. See my full comments below

- It's not clear to me why a core metabolic model was used rather than a genome-scale model. Are the computations really that limiting? I would expect that the presence of side pathways could significantly affect predictions. For example, the fact that the majority of solutions were 1 or 2 genes may be a result of already neglecting many other pathways that are present in the genome (although admittedly perhaps not typically expressed).
- The authors selected 33 genes to do combinations up to 5 genes. What was the rationale for these 33?
- They mentioned previously published strains as being excluded – but how well did the model predict the methanol coupling of growth in these cases? Were they successful positive controls?
- 6.5% of biomass is expected to be produced from methanol – is this likely to be a worthwhile bioprocess? Can this number be further increased? In the discussion (for example line 280), it seems that the authors do not want higher methanol incorporation into biomass. Why would this be the case? It seems to me that higher incorporation would lead to a greater selection pressure on methanol incorporation, which could then be repurposed for chemical production through further strain engineering.
- During the FBA simulation, why was the co-substrate concentration set to an arbitrarily high value? Would it not make more sense to take a physiological estimate from existing growth data? Does it not make a difference to the relative weighting of knockouts for growth contribution?
- In the modeling procedure, the authors remove solutions that do not grow solely on methanol (step 5). However, the resulting strains do not grow on methanol, which the authors state is expected (line 190). Could the authors clarify this apparent inconsistency?
- Would it be possible to add a laboratory evolution step to evolve the strains to higher growth rates without the plasmid being kicked out? Would the authors expect this to improve methanol incorporation, for example through the removal of any inconvenient regulatory barriers?

Reviewer #4 (Remarks to the Author):

The authors present an attempt to metabolically engineer E. coli to grow on the non-native substrate methanol using recombinant ribulose monophosphate (RuMP) pathway enzymes. They extensively use constraint based metabolic modeling (flux balance analysis) to identify gene knockout targets that don't compromise E. coli's native enzymes that are part of the RuMP pathway, but rather confer coupling between methanol assimilation and growth upon co-consumption with a multi-carbon substrate (e.g. pyruvate). Then the authors perform additional analysis to find the most promising combinations of gene deletions and co-substrates.
Then the authors chose two different strains (each with a different gene knockout configuration) to experimentally validate the in-silico analysis. The two strains indeed were able to grow only in the presence of both methanol and pyruvate in the culture media, and failed to grow on each individually, thus validating the computational analysis. The authors further characterize the incorporation of 13C labeled methanol carbon into various metabolites of RuMP pathway. The results confirm significant incorporation of 13C carbon into RuMP pathway metabolites and the extent is in high accordance with the in-silico predicted fluxes.
The manuscript provides a demonstration of a thorough and well designed implementation of constrained based metabolic modeling in the service of metabolic engineering. Synthetic methylotrophy is a prominent goal toward sustainable production of chemicals and the RuMP pathway is one of the most energy efficient pathways for methanol utilization. Therefore, it will be of value to the community both as an example for implementation of metabolic modeling and as a milestone in the race toward methylotrophy in E. coli.

- Abstract: "promising substitute for conventional food and feed competitive substrates in biotechnology". I had to read this sentence several times in order to understand the meaning. As the opening sentence the authors may want to simplify/clarify.
- Abstract: "In contrast to previous constructs", not clear what constructs means exactly. Try to clarify.
- In the abstract the authors mention that E. coli strains that solely rely on methanol as a carbon source have not yet been achieved. It might be worth to consider the exact statement in light of reference #37.
- Were the strains sequenced after the initial two passages on yeast extract? - There might be background mutations that affect the growth. This is not essential for the paper publication/acceptance.
- Figure 1. In it there are four X denoted in the same way. The resulting configuration is not a configuration by itself as far as I understand. I think it can be clearer if the Xs would have colors that would connect them to the relevant panels B-D.
- Figure 1, given its importance I think gluconate should be more prominently shown. Also it is hard to follow how it will incorporate into the central metabolism under deltaEDD pathway
- Figures 1, 3, there are various thin lines that I think are not meant to appear and make readability problematic.
- The definitions of MRR_n and MBR_n could gain from a schematic figure as a panel in the paper or as an SI figure.
- I personally find that acronyms decrease the clarity and ability to follow papers. I suggest to consider if there is a way to minimize their usage and spell out in some cases. Especially given the ease of getting confused between MRR and MMR for example. I could think of changing MRR to "methanol/R5P rate ratio" which isn't that much longer and then "normalized methanol/R5P rate ratio" which is so much clearer, but I will respect any choice of the authors.
- The term "fractional contribution" should be clearly defined in the text and not just deep in the methods.

- Consider if to change ‘gene’ to ‘reaction’ where relevant (e.g. L. 65)
- L. 62 why wasn’t glycerol included in the list of co-substrates?
- The term wild-type is first used in L. 74. It is not clear what it refers to and it should be clarified. How can a reader understand the equations if a wild type e.coli doesn’t use methanol?
- L. 140, gcdW-1, are you sure W should be capitalized?
- Figure 4, colors used in panel B have other meanings in panel A.
- L. 169 omit the word was
- L. 195 and Fig 3C&3D - could you speculate in the discussion why there is such a substantial difference in the yield between the two strains? Was the post-growth media composition analyzed using HPLC to check residual carbon source concentrations?
- L. 319 change strain to strains
- L. 425, missing spaces in units in the equation.

POINT-BY-POINT RESPONSE

We would like to thank all reviewers for the valuable comments that helped to improve the manuscript and their time and insights.

REVIEWER COMMENTS

Reviewer #1 (Remarks to the Author):

The study of Keller et al. Methanol-dependent Escherichia coli strains with a complete ribulose monophosphate cycle" is interesting to read. The methodology is convincing and the iterative process for identifying the E. coli mutants is elegant.
But in my eyes there is a conceptional issue, the authors talk about methanol as a source for biomass production. However, if I understand the metabolic mode of the mutants, there is no incorporation of methanol in the biomass.
The authors reconstruct a methanol consuming pathway in E. coli. But the biomass production relies on pyruvate. On the other hand these substrates are not serving as a nitrogen source.
The authors do also not comment about the reason for the methanol autotrophy of these mutants. The Construction of a methanol dependent ribulose phosphate cycle is interesting, but the need to decouple the glycolysis at the level of aldolase or triose phosphate isomerase counteracts biomass production clearly.

It is true that most of the biomass is formed from pyruvate; however, a non-negligible part of it is produced from methanol. Therefore, we do not see a conflict with the statement that methanol is a source for biomass production. On the contrary, we specifically aimed to identify methanol-dependent strains that incorporate methanol using a complete RuMP cycle, and we show that the strains form biomass from methanol to the extent we predicted. We rephrased the introductory part of the revised manuscript to improve clarity.

Briefly, the first studies in the field have shown that the introduction of the missing RuMP cycle genes in *E. coli* leads to the incorporation of methanol into central metabolites, but is not sufficient to achieve growth on methanol (Müller et al., 2015). One way to improve methanol assimilation efficiency is adaptive laboratory evolution. To ensure a high selection pressure towards methanol assimilation from the beginning of evolution, methanol-dependent strains are particularly promising. Such strains can also be called "methanol auxotrophs" because they can only grow when assimilating methanol. This is a consequence of an engineered metabolism, i.e. mutated in genes for enzymes in central metabolism. For a successful evolution towards growth on methanol alone, a functional methanol assimilation pathway, in this study the RuMP cycle, is crucial. In earlier methanol-dependent constructs, this criterion was not fulfilled. Specifically, in the studies the following RuMP cycle genes *rpiAB* (Meyer et al., 2018), *tktAB* or *rpiAB* (He et al., 2018), *rpe* or *rpiAB* (Chen et al., 2018) and *rpiAB* (Bennett et al., 2020) were deleted. For this reason and in contrast to the earlier publications mentioned above, we aimed to identify methanol-dependent strains with a complete RuMP cycle and where methanol can theoretically be used as the sole carbon source.

The growth rate of a methanol-dependent strain depends on two parameters: the methanol uptake efficiency and the proportion of biomass that must be stoichiometrically formed from methanol. It is expected that the methanol uptake efficiency is low without evolution, which has also been shown in several studies (Müller et al., 2015, Meyer et al., 2018, Woolston et al., 2018). Therefore, the initial growth rate of a methanol-dependent strain can only be controlled by the biomass fraction that has to be formed from methanol. If this methanol biomass fraction is too high, the initial growth rate of the methanol-dependent strain is too low and experimental implementation becomes difficult. Following

this rationale, we specifically aimed to identify methanol-dependent strains with a low methanol biomass fraction, which should ensure experimental feasibility.

By adaptive laboratory evolution, the initially low methanol biomass fraction can be increased to finally obtain a synthetic methylotrophic *E. coli* strain. This strategy was recently proven successful by Gleizer et al. in 2019 in connection with the engineering and evolution of CO_2-fixation in *E. coli*.

Regarding the nitrogen source: When we mention biomass, we are referring to biomass carbon. The source of nitrogen in all our experiments was ammonia, which is electrochemically produced from nitrogen gas, and therefore there is no need to consider alternative sources in the context of a methanol-based economy. We have made this clear in the main text in the revised version.

Concerning the decoupling of glycolysis: we do not think that the deletion of fructose-1,6-bisphosphatase (*fbp*) and triose phosphate isomerase (*tpiA*) counteracts biomass production. Fbp catalyzes a gluconeogenic reaction and its deletion should not affect glycolysis. Although the deletion of *tpiA* interrupts glycolysis, growth on methanol is still possible via the Entner-Doudoroff pathway.

Reviewer #2 (Remarks to the Author):

This is an excellent research paper that I really enjoyed reading it. I have to say that the scientific soundness, the significance of the content and the interest for the reader are high. However I am wondering if this paper fully satisfied both the criteria of novelty and advance in the field in regard to the following major point described below and previous studies in the field. This includes: one study ((doi.org/10.1038/s41589-020-0473-5) demonstrating the growth on methanol of a synthetic methylotroph and two studies (doi.org/10.1016/j.ymben.2017.11.016 & doi.org/10.1021/acssynbio.8b00093) addressing the importance of the full operation of the Rump Cycle on the methanol assimilation in synthetic methylotrophic strains. I advise the authors to discuss better those papers in regard to their results.

We are pleased to hear the reviewer found the data sound and significant. We have revised the manuscript to better distinguish our accomplishments from the ones of the publications mentioned. We agree that these are important studies in the field. The first study concerns the engineering of a synthetic methylotrophic *E. coli* that uses the reductive glycine pathway for one-carbon assimilation, which is different to the RuMP cycle used in this study. The RuMP cycle is the energetically most efficient known methanol assimilation pathway, thus, synthetic methylotrophy via the RuMP cycle is particularly promising. It also does not require elevated level of CO_2 in the culture atmosphere in contrast to the reductive glycine pathway. When considering these two points, we believe that methanol-dependent strains with a complete RuMP cycle are of high value for the scientific community, and this study clearly represents an advance towards a faster growing and biotechnologically applicable synthetic methylotrophic *E. coli*.

Major:

Point 1: The computational approach presented in this paper is interesting but I encourage the authors to perform more biological and computational validations to strengthen the novelty, the relevance and the genericity of the presented approach. For instance, does this approach reproduce the biological data obtain with previous methanol dependant strains with an incomplete RuMP cycle? Is this approach enough generic to be used to implement other non-native carbon source in E. coli? Here successful biological validations has been shown for 2 single K/O but what about the predicted double K/O? All these questions should be answered and discussed in the manuscript.

Thank you for your comment. Indeed, previously published methanol-dependent strains were identified by our approach (Supplementary Table 2a) but were filtered out due to their incomplete RuMP cycle in a second step in our study. Specifically, the strains have the following number in Supplementary Table 2a:
Strain Δ*edd*Δ*rpiAB* (Meyer et al., 2018): 5420
Strain Δ*rpiAB*Δ*zwf* (He et al., 2018): 5430
Strain Δ*tktAB*Δ*zwf* (He et al., 2018): 3684
Strain Δ*edd*Δ*pgi*Δ*rpiAB* (Bennett et al., 2020): 5264
Strain Δ*rpiAB* (Chen et al., 2018): 5419
Strain Δ*rpe* (Chen et al., 2018): 3364
The fact that all published strains were identified confirms the strength of the applied approach to predict methanol-dependent strains. We have rewritten the first paragraph of the result section to better emphasize that methanol-dependent strains with an incomplete RuMP cycle were identified but were not considered for further analysis.

With regard to the generality of the approach for the implementation of other non-native carbon sources: We see no obstacles why the approach could not be applied to other non-indigenous carbon sources. The recent studies on CO_2 fixation (Antonovsky et al., Gleizer et al) support this notion.

We agree that it would be interesting to biologically validate double knockout strains, but this would be beyond the scope of this study. They all require a large fraction of their biomass to be produced from methanol and it remains doubtful whether the initial efficiency of the synthetic methanol assimilation pathway is sufficient to support growth. The double knockout strains will become experimentally feasible once a more efficient methanol assimilation pathway over the RuMP cycle is known.

Point 2: The key point of the method presented here, i.e. the calculation of the ratios MRR and MBR is not trivial to understand. For instance, following the explanations given in both the results and methods sections, I understand that methanol influx was fixed at 1 mmol/gDCW/h and that co substrate influx varied between 0 and 1000). Based on that, I found that MRR could vary between 1 (Co substrate influx = 0, methanol influx = 1 and R5P production = 1) and 0.001 (Co substrate influx = 1000, methanol influx = 1 and R5P production = 1001 (I assume that R5P can also be produced by the co substrate, is it correct?)). Then in this case MRRn can vary between 0 and 1001 which is far to what you obtain (i.e. 0> MRRn> 1). I trust your method and I know that I definitely miss something but what? The raw data of the MRR and MBR for both the starting strain and the KO strains and eventually more explanations should be provided to help the reader.

We acknowledge, this part of the manuscript was difficult to follow. We rewrote the corresponding part to better clarify the key points of our approach. We have restructured the first and second paragraph of the result section. We also added a figure with a numerical example (Supplementary Fig. 2 and 3) and transformed the equations to calculate the methanol-derived fractions (Supplementary Fig. 2d and 3d).

Below is a summary of the equations used regarding normalized MRR:

$$(1): MRR = \frac{methanol\ influx_{strain}}{R5P\ production_{strain}}$$

$$(2): MRR_n = \frac{MRR_{methanol-dependent\ strain,methanol+co-substrate}}{MRR_{parental\ strain,methanol}}$$

Combination of (1) and (2) results in (3):

$$(3): MRR_n = \frac{\dfrac{methanol\ influx_{methanol-dependent\ strain,methanol+co-substrate}}{R5P\ production_{methanol-dependent\ strain,methanol+co-substrate}}}{\dfrac{methanol\ influx_{parental\ strain,methanol}}{R5P\ production_{parental\ strain,methanol}}}$$

$$(4): methanol\ influx_{methanol-dependent\ strain, methanol+co-substrate} = methanol\ influx_{parental\ strain, methanol} = 1\ mmol\ gcdw^{-1}\ h^{-1}$$

Combination of (3) and (4) results in (5):

$$(5): MRR_n = \frac{\frac{1\ mmol\ gcdw^{-1}\ h^{-1}}{R5P\ production_{methanol-dependent\ strain, methanol+co-substrate}}}{\frac{1\ mmol\ gcdw^{-1}\ h^{-1}}{R5P\ production_{parental\ strain, methanol}}}$$

Transformation of (5) results in (6):

$$(6): MRR_n = \frac{R5P\ production_{parental\ strain, methanol}}{R5P\ production_{methanol-dependent\ strain,, methanol+co-substrate}}$$

Equation (7) follows from the fact that the predicted methanol-dependent strains are as efficient as the parental strain (without any knockouts) in producing R5P/biomass from methanol as sole carbon source. In other words, the gene deletions that are required to couple methanol utilization to growth do not affect methanol assimilation efficiency.

$$(7): R5P\ production_{parental\ strain, methanol} = R5P\ production_{methanol-dependent\ strain, methanol}$$

Combination of (6) and (7) results in (8):

$$(8): MRR_n = \frac{R5P\ production_{methanol-dependent\ strain, methanol}}{R5P\ production_{methanol-dependent\ strain, methanol+co-substrate}}$$

$$(9): MRR_n = methanol - derived\ R5P\ fraction$$

Combination of (8) and (9) results in (10):

$$(10): methanol - derived\ R5P\ fraction = \frac{R5P\ production_{methanol-dependent\ strain, methanol}}{R5P\ production_{methanol-dependent\ strain, methanol+co-substrate}}$$

Equation (10) is now easier to understand for the reader and more suitable for the visualization of the approach (Supplementary Fig. 2 and 3). Therefore, we are using this equation rather than the ones for the MRR and the normalized MRR.

Regarding your comment about the calculation of the MRR_n: It is true that the MRR of a methanol-dependent strain can vary between a small number (methanol-independent production) and a higher number (methanol-dependent, up to 5). Number can also be above 1 as the influx of methanol of 1 mmol $gcdw^{-1}$ h^{-1} together with an excess of co-substrate can produce less than 1 mmol $gcdw^{-1}$ h^{-1} R5P in a methanol-dependent regime. To calculate MRR_n, the MRR of the methanol-dependent strain is divided by the MRR of the parental strain. The MRR of the parental strain is based on the methanol influx, which is set to 1 mmol $gcdw^{-1}$ h^{-1} and the R5P production without any additional substrates than methanol. This means that the MRR of the parental strain represents the highest possible number (5) for a MRR as its production is fully dependent on methanol. Therefore, it is a constant value. In consequence, MRR_n can only vary between 0 (methanol-independent) and 1 (fully methanol-dependent, $MRR_{methanol\text{-}dependent\ strain}$ and $MRR_{parental\ strain}$ are the same). We hope that our explanation is helpful for a better understanding of the calculations performed.

Point 3: Authors argue that "ALE to methanol-dependent ancestor strains with a complete RuMP cycle is a promising strategy" to (I guess!) establish methylotrophy in E. coli but this should be demonstrated to really represent a significant advance in the field of synthetic methylotrophy.

We set up chemostats and conducted an ALE experiment with methanol in excess and the co-substrate at limiting concentrations for the two strains Δ*fbp* and Δ*tpiA* over the time period of 2-3 months (Supplementary Fig. 6). Indeed, we observed an increase in growth rate for both strains; however, the methanol incorporation into central metabolites remained the same for strain Δ*tpiA* and decreased for strain Δ*fbp*. Importantly, we show that the methanol-dependent growth of both strains is stable over a longer time period. This is not self-evident from previous experiences and confirms the potential of the strains for a long-term evolution experiment. We thus believe that the continuation of the chemostat evolution experiments is promising but feel its running time requirement are beyond the scope of this study. When we use as a benchmark the recent study by Gleizer et al. (synthetic autotrophic *E. coli* with a rewiring pentose phosphate pathway), the time frame to observe a clear phenotype was ~1 year. We think that both cases might require a similar number of genetic adaptations (which is a strong determinant of evolution time) due to the resemblance between the Calvin and the RuMP cycle. We continue on evolving the two methanol-dependent strains and will cover the outcome in a follow-up paper.

Minor:
P2 L19-20: please add methanol in addition to the already written formate and CO2.

We have restructured the introduction to focus on synthetic methylotrophy via RuMP cycle. We now include the respective study to the discussion part of the revised manuscript.

Fig 1B: I wonder if it is biologically possible to reach a such high growth rate (i.e. 1,5 h-1). What is the co-substrate uptake rate? Please add it on the figure. Is the growth dependent to the co substrate? Please add it in the text or in the figure.

Thank you for your remark. Fig. 1 is intended to illustrate the three possible metabolic categories to which the tested strains could belong. Therefore, we had not focused on the use of physiologically relevant values. The co-substrate uptake rate was set to a value that resulted in a number dimension that allowed a direct comparison of the three numbers. Specifically, it was 50 mmol co-substrate $gcdw^{-1}$ h^{-1} in Fig. 1b, 40 in Fig. 1c, and 11 in Fig. 1d. We have now unified the co-substrate uptake rate to 10 mmol $gcdw^{-1}$ h^{-1} and added this information to the figure legend.

P5 L110: What is the genotype of the wild type strain? If it's the strain containing the full RUMP cylce, then wild type is really not appropriate. This should be changed by starting strain.

We agree. The use of wild-type in this context is incorrect. We exchanged it now with parental strain throughout the manuscript.

Fig 2A & B: please correct: To my understanding both presented ratio are the normalised one so an underscript "n" should be added.

An underscript of n should have been added, we agree. With the transformation of the formula, the figure titles have changed and the normalization is not part of them anymore.

P7 L175: Please rephrase the sentence: " …methanol dependant for…" by methanol dependant under acetate….cultivation conditions.

We modified the sentence as suggested.

Reviewer #3 (Remarks to the Author):

In this study, the authors develop methanol converting strains of E. coli based on the ribulose monophosphate cycle and utilizing flux balance analysis metabolic modeling. They experimentally built and profiled two strains, with fructose bisphosphatase and triosephosphate isomerase deletions, selected from ~1000 computationally-predicted strains that were high performing. They use a modified version of the core metabolic model from the BiGG database for the modeling component, which had the ED and RuMP cycle added. They used a recently published algorithm for predicted methanol-coupled growth designs. Experimental profiling included growth and 13C tracing measurements. The work is an interesting endeavor in computationally driven metabolic engineering, and the validation is promising. The use of a metabolic model of core metabolism only, rather than a genome-scale model, is the largest apparent practical concern, but as the authors included experimental validation, the importance of that concern is reduced. See my full comments below

- It's not clear to me why a core metabolic model was used rather than a genome-scale model. Are the computations really that limiting? I would expect that the presence of side pathways could significantly affect predictions. For example, the fact that the majority of solutions were 1 or 2 genes may be a result of already neglecting many other pathways that are present in the genome (although admittedly perhaps not typically expressed).

We had tested a genome scale model (iML1515), but it did not turn out to be useful in our context of strain design. For example, the search for knockouts that separate the upper and lower parts of glycolysis (such as the PGK knockout), is not possible by a single mutation in the genome scale model. Even a triple knockout of PGK, the ED pathway, and the methylglyoxal pathway (Δ*pgk*Δ*edd*Δ*msgA*) is viable e.g. on xylose and it is difficult to find the bypass as it passes through non-central reactions. In fact, we had to knockout 8 reactions (PGK, EDD, MGSA: DRPA, POR5, GART, FTHFLi, PAI2T) to stop the flux through glycolysis. Therefore, the minimum number of knockouts needed to find a solution to our problem is much higher and difficult to interpret. In addition, the runtime required to find each solution is longer by many orders of magnitude (both because the model is larger so each FBA simulation is slower, and because we have many more knockouts to test).
From our experience with other metabolic engineering goals (CO_2 assimilation: Antonovsky et al., 2016 and Gleizer et al., 2019; glycerate auxotrophs: Aslan et al., 2020, etc.) we have found the core model to be a good balance between fast runtimes (facilitated by its small scale) and sufficient complexity to provide reasonable results. This is demonstrated by the fact that both predicted strains tested in this work indeed were methanol-dependent, as the reviewer points out. In addition, both strains are not methanol-dependent in the iML1515 model.

- The authors selected 33 genes to do combinations up to 5 genes. What was the rationale for these 33?

The 33 possible knockouts considered in this study are essentially all possibilities that exist in the core model (in total 114 reactions), when we exclude transports (27 reactions), exchange reactions (26 reactions) and metabolically "redundant" reactions (28 reactions). The latter "redundant reactions" are referring to (i) reactions that are directly coupled to another reaction - i.e. they are in the same linear pathway with no branching point in between (e.g., the deletion of the glucose 6-phosphate dehydrogenase (ΔZWF) reaction was not considered since the deletion of the 6-phosphogluconolactonase reaction (ΔPGL) is equivalent and it is thus sufficient to test only the latter) - or (ii) isozymes that we have combined as they can be considered equivalent in the model (e.g., malic enzyme reactions ΔME1 and ΔME2). The reactions are provided in Supplementary Table 1.

- They mentioned previously published strains as being excluded – but how well did the model predict the methanol coupling of growth in these cases? Were they successful positive controls?

Thank you for your comment. Indeed, previously published methanol-dependent strains were identified by our approach (Supplementary Table 2a) but were filtered out due to their incomplete RuMP cycle in a second step. Specifically, the strains have the following number in Supplementary Table 2a:

Strain Δ*edd*Δ*rpiAB* (Meyer et al., 2018): 5420
Strain Δ*rpiAB*Δ*zwf* (He et al., 2018): 5430
Strain Δ*tktAB*Δ*zwf* (He et al., 2018): 3684
Strain Δ*edd*Δ*pgi*Δ*rpiAB* (Bennett et al., 2020): 5264
Strain Δ*rpiAB* (Chen et al., 2018): 5419
Strain Δ*rpe* (Chen et al., 2018): 3364
The fact that all published strains were identified confirms the strength of the applied approach to predict methanol-dependent strains. We have rewritten the first paragraph of the result section to better emphasize that methanol-dependent strains with an incomplete RuMP cycle were identified but were not considered for further analysis.

- 6.5% of biomass is expected to be produced from methanol – is this likely to be a worthwhile bioprocess? Can this number be further increased? In the discussion (for example line 280), it seems that the authors do not want higher methanol incorporation into biomass. Why would this be the case? It seems to me that higher incorporation would lead to a greater selection pressure on methanol incorporation, which could then be repurposed for chemical production through further strain engineering.

We agree that 6.5% may seem little. As the reviewer points out, we specifically aimed to identify methanol-dependent strains with such a degree of methanol incorporation and a complete RuMP cycle (note also the prediction for RuMP cycle intermediates is 40%). In general, strains that require a large fraction of their biomass to be built from methanol carry a high metabolic burden on the synthetic pathway from the very beginning. In other words, we aimed to identify methanol-dependent strains that are experimentally feasible for methanol-dependent growth. Such strains bear a high potential for experimental evolution and elevated methanol incorporation and full synthetic methylotrophy. The relatively low dependence on methanol in such strains (and the addition of a co-substrate) is critical in the early phase of evolution, and the conditions generated in the chemostat provide the evolutionary pressure to increase the methanol dependence gradually, up to 100% eventually. The long term goal is a fully synthetic methylotrophic *E. coli* that would form 100% of its biomass from methanol.

- During the FBA simulation, why was the co-substrate concentration set to an arbitrarily high value? Would it not make more sense to take a physiological estimate from existing growth data? Does it not make a difference to the relative weighting of knockouts for growth contribution?

FBA only considers the uptake rate of a substrate. The arbitrarily high value has been chosen to assure that the co-substrate is available in excess in all tested conditions. Most importantly, it had to be in excess in relation to the bottleneck reaction HPS, which was set to a flux of 1 mmol $gcdw^{-1}$ h^{-1}. The utilization of physiological estimates should not affect the outcome of the analysis.

- In the modeling procedure, the authors remove solutions that do not grow solely on methanol (step 5). However, the resulting strains do not grow on methanol, which the authors state is expected (line 190). Could the authors clarify this apparent inconsistency?

We agree that this is an apparent inconsistency. We have reworded the main text to better explain this point. According to FBA, the introduction of the three RuMP cycle genes *mdh*, *hps* and *phi* into *E. coli* should allow for growth on methanol as sole carbon source, but does not do so in reality, i.e. experimentally, as has been shown in a number of previous studies (Müller et al., 2015, Bennett et al., 2018, Meyer et al. 2018, Woolston et al., 2018). Indeed, the introduction of the genes led to methanol incorporation into central metabolites but was not sufficient to achieve growth on methanol. It is not uncommon to observe that metabolic models are "imperfect" to predict actual growth. They might fail due to wrongly identified reactions, incomplete networks, substrate affinities or regulation, i.e. the enzyme is not produced under the tested conditions.

Since the organism is not able to recapitulate the predicted flux from the model and use methanol as sole source of carbon and energy, a co-substrate is necessary to set up an evolution experiment. In such an experiment random mutations (not predicted in the model) can occur which give the organism a growth advantage. However, if methanol is used in addition to a co-substrate, evolution will drive the population to grow better on the co-substrate instead of methanol and methanol incorporation will eventually vanish. To ensure a high selection pressure towards efficient methanol incorporation from the beginning of the evolution, a strain background that allows small but significant methanol incorporation is particularly promising. Previously published methanol-dependent strains grew in the presence of methanol but did not contain a complete RuMP cycle as a result of mutations in *rpiAB* (Meyer et al., 2018), *tktAB* or *rpiAB* (He et al., 2018), *rpe* or *rpiAB* (Chen et al., 2018) and *rpiAB* (Bennett et al., 2020). These strains thus allow methanol incorporation and may improve during evolution; however, they do not have the potential to ultimately grow on methanol as sole carbon source. The proposed strains from this publication do have this potential and are ideal ancestor strains for evolution. Overall, the long term goal would be to evolve one of our proposed methanol-dependent strains into a fully synthetic methylotrophic *E. coli*.

- Would it be possible to add a laboratory evolution step to evolve the strains to higher growth rates without the plasmid being kicked out? Would the authors expect this to improve methanol incorporation, for example through the removal of any inconvenient regulatory barriers?

The methanol-dependent growth ensures that plasmids containing the RuMP cycle genes cannot be kicked out. To prove this and to evolve the strains, we set up chemostats and conducted an ALE experiment with methanol in excess and the co-substrate at limiting concentrations for the two strains Δ*fbp* and Δ*tpiA* over the time period of 2-3 months (Supplementary Fig. 6). Indeed, we observed an increase in growth rate; however, the methanol incorporation into central metabolites remained the same for strain Δ*tpiA* and decreased for strain Δ*fbp* (see also our comments to reviewer 2).
The short-term evolution experiment has shown that the growth rate improved for the identified methanol-dependent strains and that the methanol-dependent growth of both strains is stable over a longer time period. This is not self-evident from previous experiences and confirms the potential of the strains for a long-term evolution experiment. We thus believe that the continuation of the chemostat evolution experiments is promising but feel its running time requirement are beyond the scope of this study. When we use as a benchmark the recent study by Gleizer et al. (synthetic autotrophic *E. coli* with a rewiring pentose phosphate pathway) the time frame to observe a clear phenotype was ~1 year. We think that both cases might require a similar number of genetic adaptations (which is a strong determinant of evolution time) due to the resemblance between the Calvin to the RuMP cycle. We continue on evolving the two methanol-dependent strains and will cover the outcome in a follow-up paper.

Reviewer #4 (Remarks to the Author):

The authors present an attempt to metabolically engineer E. coli to grow on the non-native substrate methanol using recombinant ribulose monophosphate (RuMP) pathway enzymes. They extensively use constraint based metabolic modeling (flux balance analysis) to identify gene knockout targets that don't compromise E. coli's native enzymes that are part of the RuMP pathway, but rather confer coupling between methanol assimilation and growth upon co-consumption with a multi-carbon substrate (e.g. pyruvate). Then the authors perform additional analysis to find the most promising combinations of gene deletions and co-substrates.
Then the authors chose two different strains (each with a different gene knockout configuration) to experimentally validate the in-silico analysis. The two strains indeed were able to grow only in the presence of both methanol and pyruvate in the culture media, and failed to grow on each individually, thus validating the computational analysis. The authors further characterize the incorporation of 13C labeled methanol carbon into various metabolites of RuMP pathway. The results confirm significant

incorporation of 13C carbon into RuMP pathway metabolites and the extent is in high accordance with the in-silico predicted fluxes.
The manuscript provides a demonstration of a thorough and well designed implementation of constrained based metabolic modeling in the service of metabolic engineering. Synthetic methylotrophy is a prominent goal toward sustainable production of chemicals and the RuMP pathway is one of the most energy efficient pathways for methanol utilization. Therefore, it will be of value to the community both as an example for implementation of metabolic modeling and as a milestone in the race toward methylotrophy in E. coli.

- Abstract: "promising substitute for conventional food and feed competitive substrates in biotechnology". I had to read this sentence several times in order to understand the meaning. As the opening sentence the authors may want to simplify/clarify.

We rephrased the sentence as suggested.

- Abstract: "In contrast to previous constructs", not clear what constructs means exactly. Try to clarify.

We rephrased this sentence.

- In the abstract the authors mention that E. coli strains that solely rely on methanol as a carbon source have not yet been achieved. It might be worth to consider the exact statement in light of reference #37.

We rephrased this sentence.

- Were the strains sequenced after the initial two passages on yeast extract? - There might be background mutations that affect the growth. This is not essential for the paper publication/acceptance.

Thank you for your comment. Indeed, it would be interesting to understand the required adaptations for the omission of the yeast extract from the growth medium. We propose sequencing strains after a long term evolution experiment.

- Figure 1. In it there are four X denoted in the same way. The resulting configuration is not a configuration by itself as far as I understand. I think it can be clearer if the Xs would have colors that would connect them to the relevant panels B-D.

We have adapted the figure accordingly.

- Figure 1, given its importance I think gluconate should be more prominently shown. Also it is hard to follow how it will incorporate into the central metabolism under deltaEDD pathway

We have added a second entry point of gluconate on the left side of the figure.

- Figures 1, 3, there are various thin lines that I think are not meant to appear and make readability problematic.

Indeed, this is probably due to the file format that was used for the manuscript. We adapted the figure presentation.

- The definitions of MRR_n and MBR_n could gain from a schematic figure as a panel in the paper or as an SI figure.

We have tried to clarify the key points of our method by spelling out MBR and MRR to methanol-derived biomass fraction and methanol-derived R5P fraction, respectively. Furthermore, we have restructured the first and second paragraph of the result section, added a figure with a numerical example

(Supplementary Fig. 2 and 3) and transformed the equation to calculate the methanol-derived fractions (Supplementary Fig. 2d and 3d, Equation (1) and (2)).

- I personally find that acronyms decrease the clarity and ability to follow papers. I suggest to consider if there is a way to minimize their usage and spell out in some cases. Especially given the ease of getting confused between MRR and MMR for example. I could think of changing MRR to “methanol/R5P rate ratio” which isn’t that much longer and then “normalized methanol/R5P rate ratio” which is so much clearer, but I will respect any choice of the authors.

We rewrote this section and now spell out MBR and MRR as methanol-derived biomass and R5P fraction, respectively. We added a figure with a numerical example (Supplementary Fig. 2 and 3) and transformed the equation to calculate the methanol-derived fractions (Supplementary Fig. 2d and 3d). For the detailed transformation of the equation, see answer to reviewer 2.

- The term “fractional contribution” should be clearly defined in the text and not just deep in the methods.

We exchanged "fractional contribution" by "labeled fraction" throughout the manuscript.

- Consider if to change ‘gene’ to ‘reaction’ where relevant (e.g. L. 65)

We checked all "gene" words and changed them to "reaction" where appropriate.

- L. 62 why wasn’t glycerol included in the list of co-substrates?

Thank you for the suggestion, we have added glycerol to the list of co-substrates. This yielded an additional number of 214 methanol-dependent strains.

- The term wild-type is first used in L. 74. It is not clear what it refers to and it should be clarified. How can a reader understand the equations if a wild type e.coli doesn’t use methanol?

We agree that the expression "wild-type strain" is not ideal and exchanged it by "parental strain" throughout the manuscript.

- L. 140, gcdW-1, are you sure W should be capitalized?

We exchanged gcdW by gcdw throughout the manuscript.

- Figure 4, colors used in panel B have other meanings in panel A.

Thank you for your suggestion to improve the figure. We changed the colors in panel B to make the graph more consistent.

- L. 169 omit the word was

We have restructured this paragraph.

- L. 195 and Fig 3C&3D - could you speculate in the discussion why there is such a substantial difference in the yield between the two strains? Was the post-growth media composition analyzed using HPLC to check residual carbon source concentrations?

We have checked the supernatant of stationary phase cultures of both strains Δ*fbp* and Δ*tpiA* (ancestral and evolved) for residual pyruvate concentration and could not detect any (Supplementary Fig. 5). We could also not detect accumulating metabolites with the applied method (UV-Vis detector, 190 nm). Therefore, we conclude that both strains are able to utilize the entire amount of provided pyruvate and the difference in yield originates from a different flux distribution of the two strains. This could be

explained by an increased CO2 production in strain Δ*fbp* since no other significant product formation was observed after complete pyruvate consumption. Potentially, the pyruvate flux into the TCA cycle might be higher in the strain Δ*fbp* than in the strain Δ*tpiA*. This would lead to a proportionally higher loss of carbon in the form of CO_2 in the strain Δ*fbp* and could explain the difference in yield. The same might be true for the ancestral and evolved strain Δ*tpiA*, which also had a yield difference. We added these observations to the manuscript.

- L. 319 change strain to strains

Corrected.

- L. 425, missing spaces in units in the equation.

Corrected.

Reviewer #1 (Remarks to the Author):

I agree with the comments of the authors.

Reviewer #2 (Remarks to the Author):

Most of my previous comments have been addressed appropriately and answers are convincing. I still believe the strength of the manuscript could be improved through the generation of evolved strain able to growth on pure methanol as recently demonstrating in this paper (https://www.cell.com/cell/fulltext/S0092-8674(20)30875-8). However I understand that the ALE experiment is still running and the results will be shown in a follow-up paper. In my opinion, the manuscript is now acceptable if the mentioned paper, demonstrating the growth on pure methanol of a synthetic E. coli expressing the Rump pathway, is included and discussed in the revised article.

Reviewer #3 (Remarks to the Author):

The authors have sufficiently addressed my concerns. I might just suggest that the authors add some of their interesting perspective on the use of the core vs full model into the discussion section, since this may be of interest to others trying to emulate their work.

Reviewer #4 (Remarks to the Author):

The authors responded adequately to all our comments.
The corrections made for the revised manuscript improve the overall quality (especially the readability) of the work and make it publication-worthy in Nat. Comm.

Some minor points we noticed in further reading that probably should be addressed in preparing the MS for publication:

Figure 1b - the purple arrow should probably be diagonal and not horizontal.

The authors added glycerol as a possible co-substrate in their analysis but don't mention it in lines 63-64.

Lines 101-102 - the authors refer to figure 2a while they write something that is related to figure 2b.

Line 108 - the authors refer to figure 2b while they write something that is related to figure 2a. Perhaps it is worth reversing the order of the figures (2a with 2b) to be compatible with the order they appear in the text.

There is no reference to Supplementary figures 2&3 anywhere in the text, while it clearly should appear in the 'Evaluation of methanol-dependent strains in silico' results section.

Line 155 - the reference is to the wrong Supplementary figure - should be figure 4 instead of 3.

POINT-BY-POINT RESPONSE

We would like to thank all reviewers for the valuable comments that helped to improve the manuscript and their valuable time and insights.

REVIEWER COMMENTS

Reviewer #1 (Remarks to the Author):

I agree with the comments of the authors.

Thank you again for your valuable comments that significantly improved our manuscript.

Reviewer #2 (Remarks to the Author):

Most of my previous comments have been addressed appropriately and answers are convincing. I still believe the strength of the manuscript could be improved through the generation of evolved strain able to growth on pure methanol as recently demonstrating in this paper (https://www.cell.com/cell/fulltext/S0092-8674(20)30875-8). However I understand that the ALE experiment is still running and the results will be shown in a follow-up paper. In my opinion, the manuscript is now acceptable if the mentioned paper, demonstrating the growth on pure methanol of a synthetic E. coli expressing the Rump pathway, is included and discussed in the revised article.

Thank you for your input. We have adapted our statements in the abstract, introduction and discussion sections in light of the manuscript that was published by Chen et al. Furthermore, we have added a paragraph that describes the paper and its relation to our manuscript to the discussion section.

Reviewer #3 (Remarks to the Author):

The authors have sufficiently addressed my concerns. I might just suggest that the authors add some of their interesting perspective on the use of the core vs full model into the discussion section, since this may be of interest to others trying to emulate their work.

Thank you for your input.

Reviewer #4 (Remarks to the Author):

The authors responded adequately to all our comments.
The corrections made for the revised manuscript improve the overall quality (especially the readability) of the work and make it publication-worthy in Nat. Comm.

Some minor points we noticed in further reading that probably should be addressed in preparing the MS for publication:

Figure 1b - the purple arrow should probably be diagonal and not horizontal.

Thank you for pointing this out. We have adapted the figure accordingly.

The authors added glycerol as a possible co-substrate in their analysis but don’t mention it in lines 63-64.

Thank you for pointing this out. We have added glycerol to the sentence.

Lines 101-102 - the authors refer to figure 2a while they write something that is related to figure 2b.

Thank you for pointing this out. We have adapted figure 2 accordingly.

Line 108 - the authors refer to figure 2b while they write something that is related to figure 2a. Perhaps it is worth reversing the order of the figures (2a with 2b) to be compatible with the order they appear in the text.

Thank you for pointing this out. We have adapted figure 2 accordingly.

There is no reference to Supplementary figures 2&3 anywhere in the text, while it clearly should appear in the ‘Evaluation of methanol-dependent strains in silico’ results section.

Thank you for pointing this out. We have added the references to the two figures to the ‘Evaluation of methanol-dependent strains in silico’ results section.

Line 155 - the reference is to the wrong Supplementary figure - should be figure 4 instead of 3.

Thank you for pointing this out. We have corrected the sentence accordingly.
